# Electrochemical alcohols oxidation mediated by *N*-hydroxyphthalimide on nickel foam surface

**DOI:** 10.1038/s41598-020-75397-8

**Published:** 2020-11-09

**Authors:** Leila Behrouzi, Robabeh Bagheri, Mohammad Reza Mohammadi, Zhenlun Song, Petko Chernev, Holger Dau, Mohammad Mahdi Najafpour, Babak Kaboudin

**Affiliations:** 1 Department of Chemistry, Institute for Advanced Studies in Basic Sciences (IASBS), 45137-66731 Zanjan, Iran; 2 School of Physical Science and Technology, College of Energy, Soochow Institute for Energy and Materials Innovations and Key Laboratory of Advanced Carbon Materials and Wearable Energy Technologies of Jiangsu Province, Soochow University, Suzhou, 215006 China; 3 Department of Physics, University of Sistan and Baluchestan, 98167-45845 Zahedan, Iran; 4 Surface Protection Research Group, Surface Department, Ningbo Institute of Materials Technology and Engineering, Chinese Academy of Sciences, 519 Zhuangshi Road, Ningbo, 315201 China; 5 Department of Chemistry-Ångströmlaboratoriet, Uppsala University, Lägerhyddsvägen 1, 75120 Uppsala, Sweden; 6 Fachbereich Physik, Freie Universität Berlin, Arnimallee 14, 14195 Berlin, Germany; 7 Center of Climate Change and Global Warming, Institute for Advanced Studies in Basic Sciences (IASBS), 45137-66731 Zanjan, Iran; 8 Research Center for Basic Sciences and Modern Technologies (RBST), Institute for Advanced Studies in Basic Sciences (IASBS), 45137-66731 Zanjan, Iran

**Keywords:** Heterogeneous catalysis, Chemistry, Catalysis

## Abstract

Alcohol to aldehyde conversion is a critical reaction in the industry. Herein, a new electrochemical method is introduced that converts 1 mmol of alcohols to aldehydes and ketones in the presence of *N*-hydroxyphthalimide (NHPI, 20 mol%) as a mediator; this conversion is achieved after 8.5 h at room temperature using a piece of Ni foam (1.0 cm^2^) and without adding an extra-base or a need for high temperature. Using this method, 10 mmol (1.08 g) of benzyl alcohol was also successfully oxidized to benzaldehyde (91%) without any by-products. This method was also used to oxidize other alcohols with high yield and selectivity. In the absence of a mediator, the surface of the nickel foam provided oxidation products at the lower yield. After the reaction was complete, nickel foam (anode) was characterized by a combination of scanning electron microscopy (SEM), energy-dispersive X-ray spectroscopy (EDX), X-ray diffraction (XRD), X-ray absorption spectroscopy (XAS), X-ray photoelectron spectroscopy (XPS), and spectroelectrochemistry, which pointed to the formation of nickel oxide on the surface of the electrode. On the other hand, using other electrodes such as Pt, Cu, Fe, and graphite resulted in a low yield for the alcohol to aldehyde conversion.

## Introduction

Electrochemistry is one of the promising methods of molecular interactions that has attracted many chemists from the subfields of organic chemistry such as synthesis of organic compounds, oxidation, and reduction reactions^1^. The benefits of this method are high compatibility with different functional groups through controlling potentials and the low temperatures (mostly room temperature) at which reactions occur. Thus, it is a novel area for most chemical reactions^2–6^. Electrochemical oxidation of materials is a suitable alternative to chemical methods; especially on a large scale, the amount of waste generated is much less than traditional chemical methods. Moreover, electrochemical oxidation of alcohols has widespread use in fuel cells, pharmaceutical industries, and chemical synthesis^7–10^.

Generally, electrochemical oxidation is carried out either directly or indirectly. The direct oxidation method requires a high potential, and most of the functional groups are activated at high potentials^11^. To solve this problem, the indirect method was introduced. The oxidation potentials of methanol and toluene show that the oxidation of methanol is more difficult than the oxidation of toluene. However, the oxidation of benzyl alcohols is significantly easier than the oxidation of aliphatic alcohols^12^. Thus, most aliphatic alcohols could be used as solvents under electrochemical conditions due to their stability. In 1979, Shono et al. employed iodine as a base and mediator in galvanostatic conditions. Using this system, primary and secondary alcohols were oxidized to the related carboxylic acids and ketones^13^. The alkali metal nitrates are the next category of mediators for oxidation of aliphatic alcohols and various derivatives of benzyl alcohol^14,15^.

*N*-oxyl compounds such as 2,2,6,6-tetramethylpiperidine *N*-oxyl (TEMPO) and phthalimide *N*-oxyl (PINO) could be used as catalysts or mediators for the selective oxidation of organic molecules^16–18^. TEMPO and N‑hydroxyphthalimide (NHPI) are significantly different with respect to stability, price, and synthesis process. NHPI can be synthesized from phthalic anhydride and hydroxylamine through a simple method; also, NHPI (1 g≈3.26 Eur) is inexpensive compared to TEMPO (1 g≈ 23.9 Eur) and other nitroxyl radicals. However, NHPI must be converted to PINO to become reactive, but TEMPO is stable under ambient temperature^19^. In some electrochemical methods, reactive oxidants such as oxoammonium and imidoxyl species are produced from aminoxyls and *N*-hydroxyimides. In such methods, the mediator is first oxidized on the surface of the electrode, and then it oxidizes the organic materials in the solution. It has been shown that imidoxyl radicals can also be efficient mediators for hydrogen-atom transfer (HAT) from weak C–H bonds^20–23^.

The first electrochemical oxidation with TEMPO and other nitroxyl radicals was reported in 1983^24^. So far, there have been many reports of various nitroxyl radicals being used in oxidation reactions^25^. For instance, Azabicyclo-*N*-oxyl and TEMPO were investigated as catalysts for the electrochemical oxidation of sterically hindered secondary alcohols^26^. However, TEMPO and other nitroxyl groups have a very low solubility in polar environments. To solve this problem, a double system of nitroxides and halide has been applied^27^. Tanakaʼs group reported that the addition of NaBr in presence of nitroxyl mediators as a double mediatory system was useful in resolving secondary benzyl alcohols in biphasic media^28^. In 2016, a copper/TEMPO catalytic system was designed with alcohol oxidation at a lower potential and higher reaction rate than the TEMPO-alone system^29^.

To the best of our knowledge, there are few studies that have made use of nickel foam electrodes for alcohol oxidation; however, the application of nickel oxides in aqueous alkaline media is a very innovative and reliable method for the oxidation of alcohols to the related carboxylic acids^30–32^. The basis of these heterogeneous reactions is the transfer of electrons from the surface of nickel oxide to the substrate. During this reaction, a black layer of nickel(lll)(hydr)oxide is formed on the surface of the electrode^33^. In most of the approaches to preparing nickel oxide on nickel foam, a hydrothermal method is used that requires a special nickel salt source and is performed at high temperatures^34^. Another method is using alkaline media, in which the presence of an external base is essential and stopping the oxidation in the aldehyde stage is impossible^30–32^.

Recently, several studies have reported that NHPI serves as a successful co-catalyst in the aerobic oxidation reaction of organic compounds. Among a number of catalysts used, complexes of Co and Mn, as well as V, Cu, and other metals have shown a positive effect on catalytic activity^35^. Although these processes have high conversion efficiency, there are significant drawbacks such as the toxicity of the homogenized metal catalysts and the need for completing the reaction at high temperatures and under pressure^36^. Therefore, replacing them with environmentally friendly methods is a challenge.

Since there are only a few reports of using NHPI in electrochemical oxidation^37–43^, and no report of using it on the surface of nickel foam, in this study, a mild procedure to oxidize various alcohols using a mediator and Ni surface is introduced.

## Results and discussion

To attempt the oxidation of benzyl alcohol to benzaldehyde at room temperature, an electrochemical procedure was carried out using several electrodes as anodes and cathodes. It was found that the presence of a mediator was critical for the quick transfer of electrons from an electrode to a substrate under the experimental conditions. The inclusion of a mediator was necessary to decrease the potential for the oxidation reaction and increase the selectivity^17^. Three mediators were investigated: TEMPO, *N*-Hydroxysuccinimide (NHS) and NHPI. Among them, NHPI showed the best results toward alcohol oxidation. Following the selection of the mediator, various electrodes were examined to optimize the conditions for alcohol oxidation.

**Figure 1:**
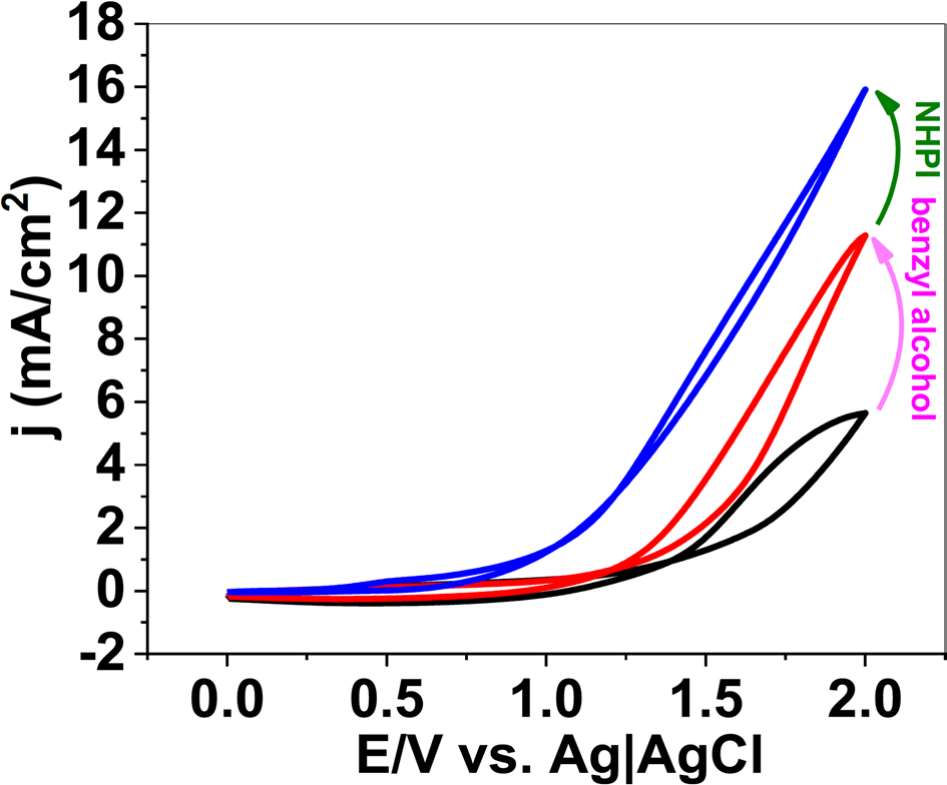
Cyclic voltammetry (scan rate 100 mV/s) of lithium perchlorate (120 mM) in the pure acetonitrile (black), addition of benzyl alcohol (200 mM) (red), and benzyl alcohol (200 mM) in the presence of NHPI (40 mM) (blue).

**Figure 2:**
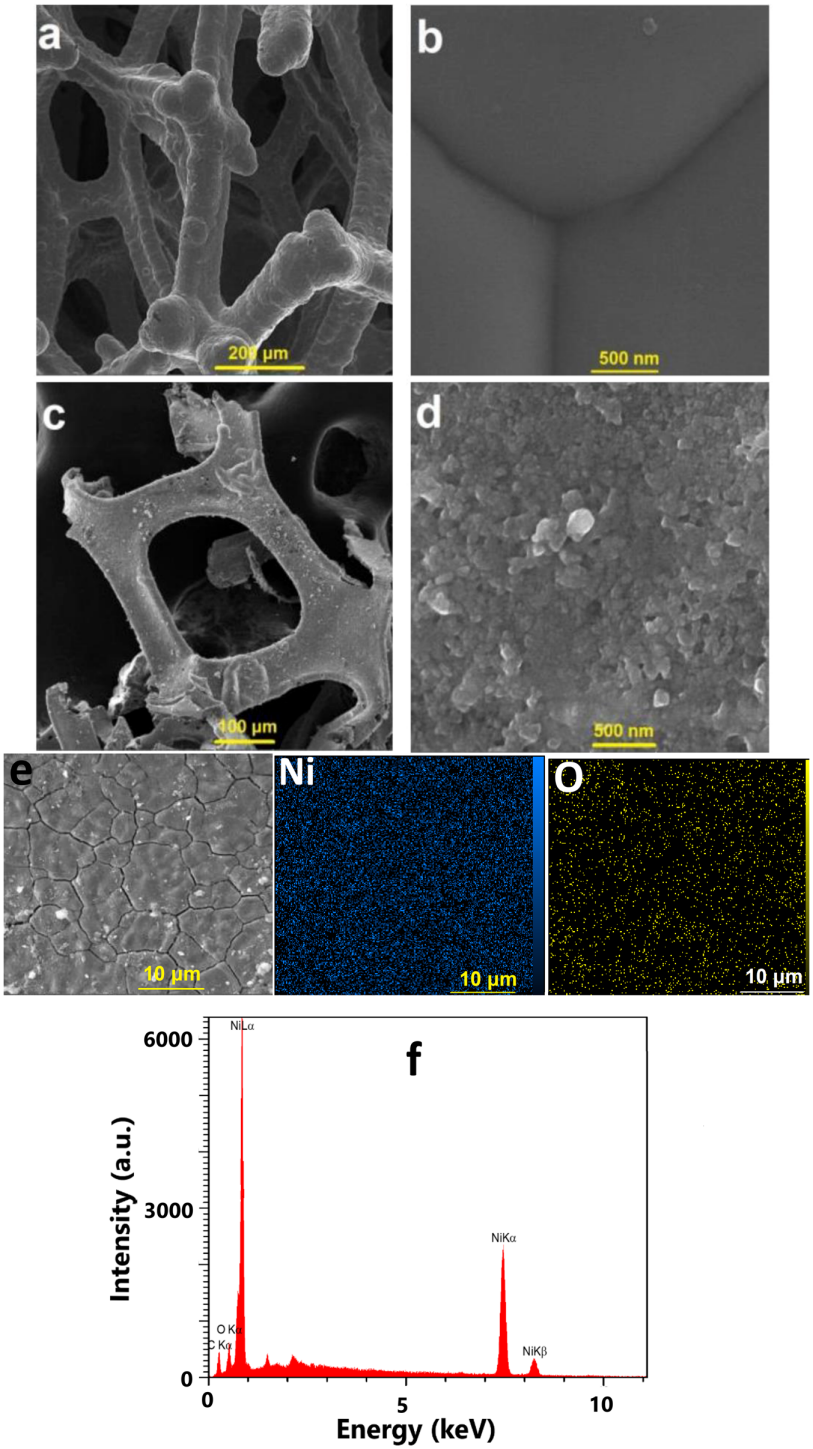
SEM images from the commercial Ni foam **(a,b)** and Ni foam(anode) after the electrochemical oxidation of 200 mM of benzylalcohol **(c,d)**. SEM–EDX mapping of the Ni foam after the electrochemical oxidation of 200 mM of benzyl alcohol **(e)**. EDX spectrum of the Ni foam after the electrochemical reaction **(f)**.

**Figure 3:**
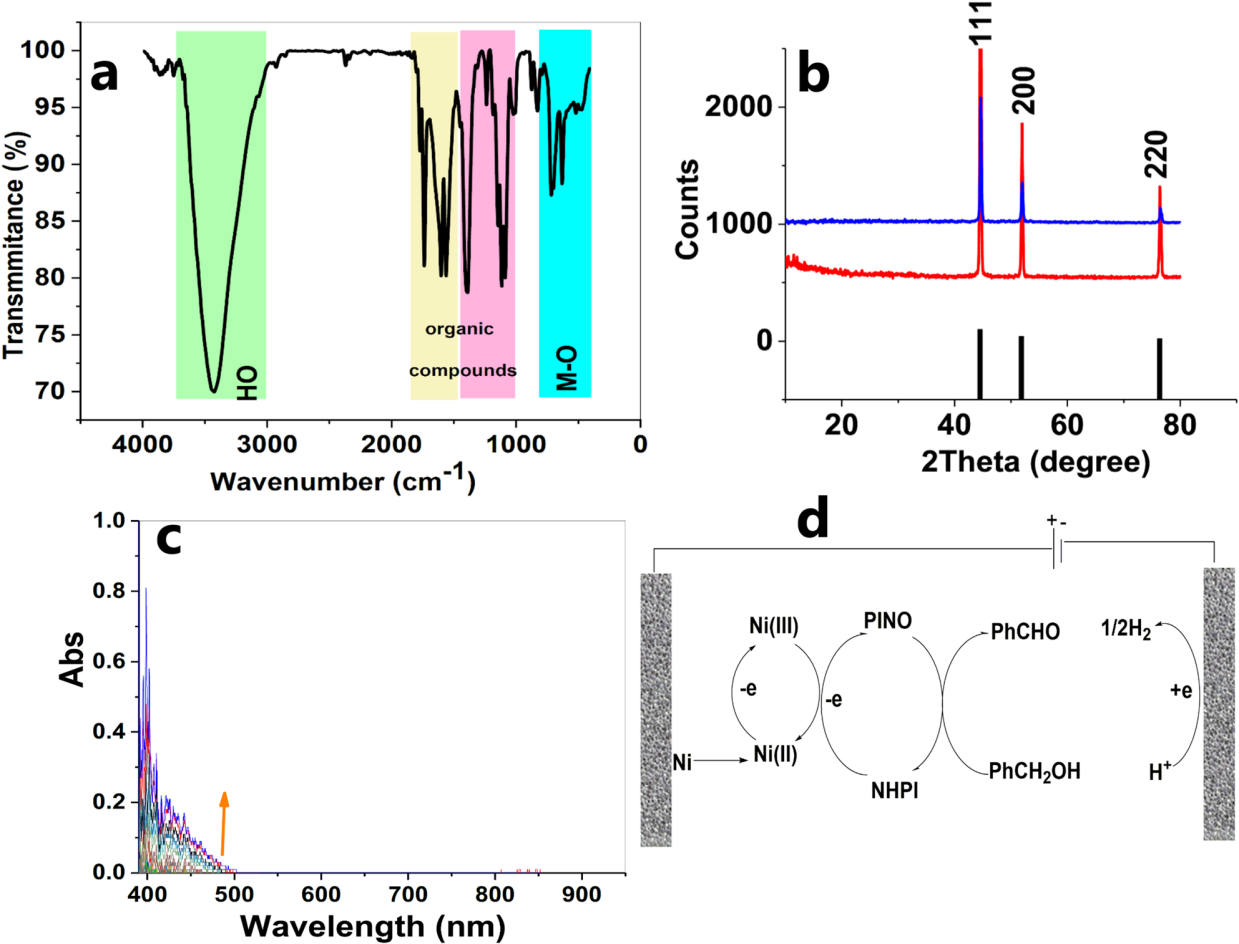
FTIR spectrum of the mechanically separated black solid on the surface of the Ni foam after the electrochemical oxidation of benzyl alcohol. Metal shavings mixed with KBr and used for FTIR analysis **(a)**. Compare XRD patterns of metallic Ni (black), commercial Ni foam as a reference (red) and Ni foam (anode) after the electrochemical oxidation of benzyl alcohol(blue) **(b)**. The spectroelectrochemical spectrum of the Ni foam in the presence of lithium perchlorate (120 mM), NHPI (40 mM) and benzyl alcohol (200 mM) at room temperature and E = 4.0 V **(c)**. A schematic image to show a proposed mechanism for alcohol oxidation **(d)**.

Although the mechanism of alcohol oxidation was not studied, the cyclic voltammetry (CV) studies were carried out with a conventional three-electrode setup in which Ni foam, Ag|AgCl|KCl_sat_ and Ni foam served as the working, reference and auxiliary electrodes, respectively. CV indicated that after the addition of benzyl alcohol to the acetonitrile, a doubling of the current density occurred, which was attributed to alcohol-oxidation reaction (Fig. 1). After adding NHPI, a further increase in the current density was observed. The metal foams were shown to have a cellular structure with high porosity and a large volume fraction of gas-filled pores. These pores could be sealed, or could form an interconnected network. The cellular structure for the nickel foam is shown in Fig. 2a,b. After the oxidation reaction, significant corrosion was observed on the surface of Ni foam (Fig. 2c,d). The SEM images indicated flake-like morphology and nanoparticles with ca. 20–100 nm on the surface of the electrode were observed. The SEM–EDX images revealed oxygen on the surface of the foam (Fig. 2e,f). FTIR spectroscopy is a helpful method for identification of M–O bonds in the metal oxides^44^. The FTIR spectra of the solids on the surface of the Ni foam after the oxidation reaction are shown in Fig. 3a. The spectra show a stretching vibration of the Ni–O octahedra at 630 and 711 cm^-1^. The peaks at 1124–1750 cm^-1^ correspond to the organic groups on the surface of the Ni foam. FTIR spectra also showed a broad peak at around 3000–3600 cm^-1^ attributed to *anti-*symmetric and symmetric O–H stretching modes.

The XRD patterns indicate that only metallic Ni was present before and after the electrochemical reaction (Fig. 3b). We, therefore, conclude that the amount of NiO_x_ on the surface of the foam is small and it is amorphous.

In the next step, we used in-situ visible spectroscopy, which was measured using a 1.0 cm^2^ spectroelectrochemical cell (Fig. S5) and a three- electrode setup. 4.0 V (without ohmic-drop correction) was applied and visible absorption of the electrode was recorded every 10 min. The broad peak at 400–500 nm is related to Ni(III)/(IV)(hydr) oxide (Fig. 3c). As Ni(III) or (IV) ions act as strong oxidants, we hypothesize that these ions play an important role in the alcohol-oxidation reaction. All these experiments could show that the high-valent Ni(III) or (IV) formed under the potential could oxidize NHPI to PINO. In the next step, PINO oxidized alcohol to aldehyde (Fig. 3d).

**Figure 4:**
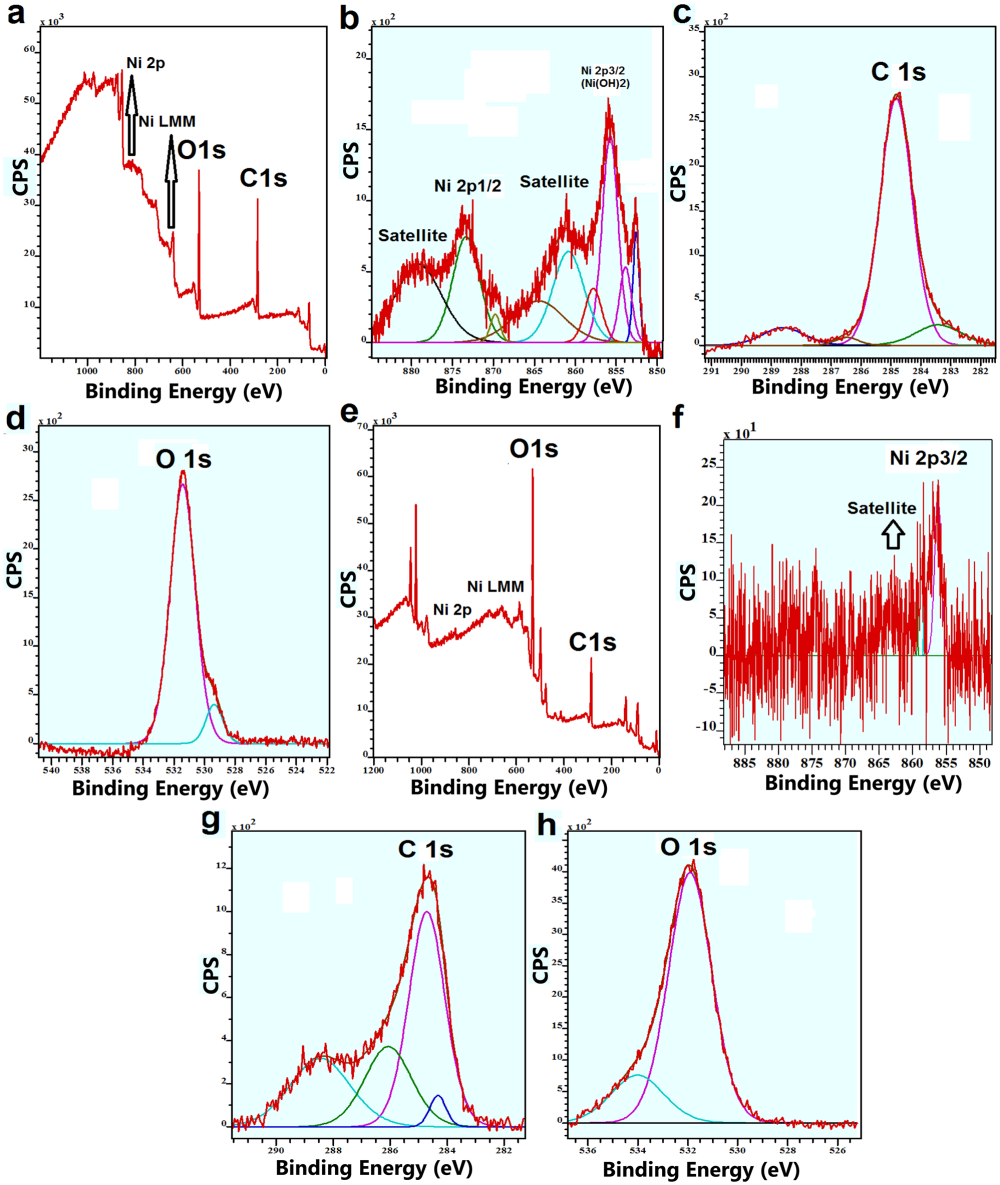
XPS spectra for the commercial Ni-foam **(a–d)** and Ni foam after **(e–h)** oxidation of benzyl alcohol (200 mM) in the solution of lithium perchlorate as an electrolyte, and NHPI (40 mM) as a mediator.

O, C, and Ni were detected on the surface of the Ni foam before the reaction by XPS (Fig. 4a). The peaks for the Ni2p region have significantly split spin–orbit components (18.0 eV). Ni2p_3/2_ for the foam showed two peaks at 852.5 and 855.9 eV, which were attributed to metallic Ni and Ni(OH)_2_, respectively (Fig. 4b)^45^. Ni2p_1/2_ for the foam showed a weak peak at 873.5 eV (Fig. 4b). Ni satellites for Ni2p_3/2_ and Ni2p_1/2_ could also be observed at 861 and 879 eV, respectively, indicating the presence of Ni(II) hydroxide^45^. It seems that the carbon in XPS equipment covers the sample, which was observed at 284.4 eV related to C–C (Fig. 4c). The designated area for O1s showed different peaks attributed to OH_2_, adsorbed OH, and O on the surface of the electrode (Fig. 4d).

On the other hand, O, C, and Ni were detected on the surface of the Ni foam by XPS, following the reaction (Fig. 4e). Ni2p_3/2_ for the foam after the reaction showed a broad peak at 856–858 eV, which was attributed to Ni(II) and Ni(III) oxides (Fig. 4f)^45^. The observed carbon area suggests that in addition to carbon from the equipment, other carbon compounds containing C–O and C=O could cover the Ni foam following the reaction (Fig. 4g). The designated area for O1s showed different peaks attributed to OH_2_, adsorbed OH, and O on the surface of the foam after the reaction (Fig. 4h).

**Figure 5:**
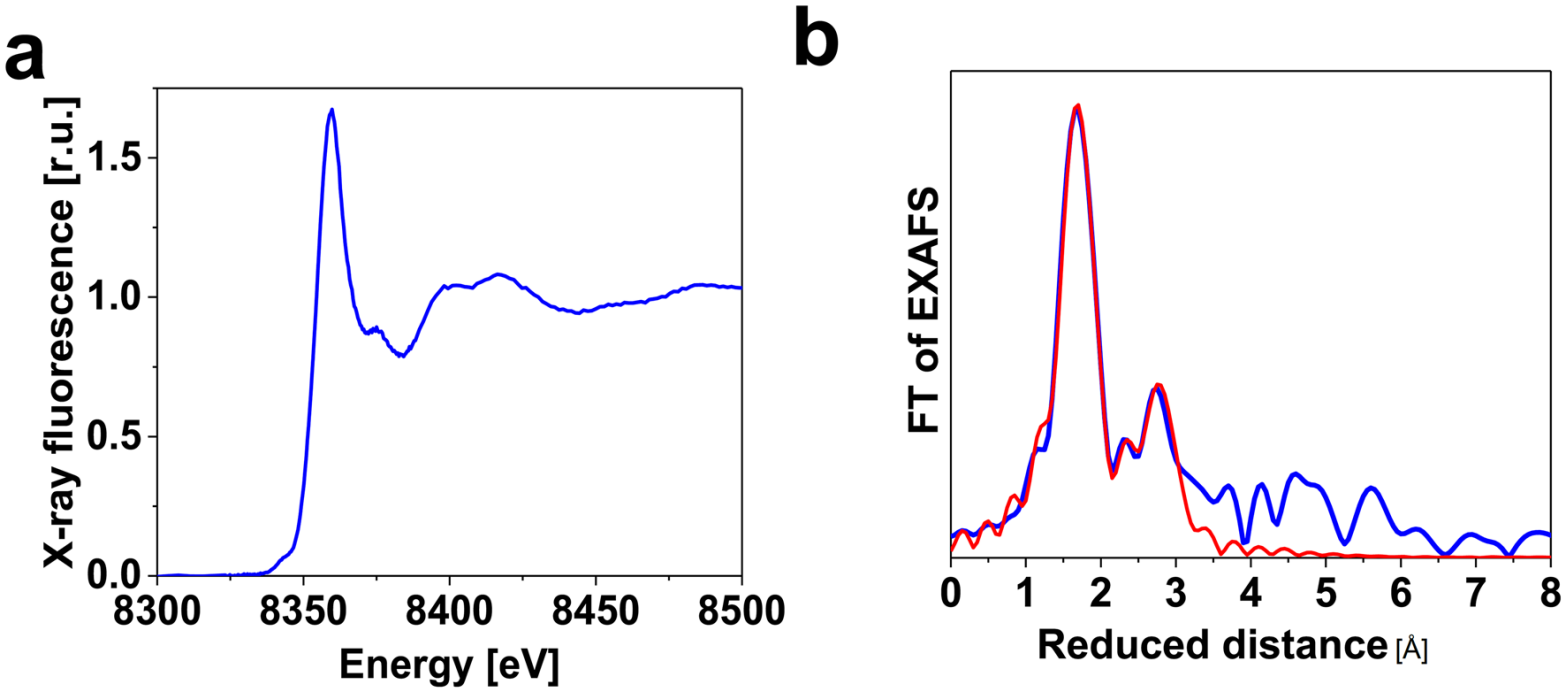
XANES spectra **(a)** and Fourier-transform of the EXAFS spectra **(b)** of the Ni foam after the electrochemical oxidation of benzyl alcohol at 4 V for 8:30 h in lithium perchlorate (120 mM) , NHPI (40 mM) and benzyl alcohol (200 mM, pH = 5). The blue and red lines show the experimental data and simulation, respectively. The k^3^-weighted EXAFS oscillations are shown in Fig. S6. The fit parameters for the simulations are given in Table 1.

X-ray absorption spectroscopy (XAS) was performed to obtain information about the oxidation state and structure of Ni foam after the reaction. XANES spectra show that after the reaction, the foam had a higher oxidation state (2.1) than metallic nickel (Fig. 5a).

The simulation results of Fourier transformed EXAFS-spectra of Ni foam after the reaction show a high main Ni–O peak that can be simulated by two Ni–O shells at 2.04 Å and 2.45 Å (Fig. 5b, Table 1). The spectrum has a Ni–Ni peak corresponding to di-μ-oxo-bridged Ni, which could be simulated by three Ni–Ni shells at 2.77 Å, 2.96 Å and 3.11 Å.

**Table 1:** Parameters obtained by the simulation of the k^3^-weighted EXAFS spectra shown in Fig. 5.

Sample		*Ni-O*_*short*_	*Ni-O*_*long*_	*Ni-Ni*_*short*_	*Ni–Ni*	*Ni-Ni*_*long*_
Ni foam after the reaction	Distance (Å)	2.04 ± 0.01	2.45 ± 0.02	2.77 ± 0.02	2.96 ± 0.04	3.11 ± 0.03
	Coordination number	9.2 ± 1.2	4.5 ± 1.3	1.5 ± 0.6	2.1 ± 1.1	2.1 ± 0.8

The energy shift (ΔE_0_, 1.4 eV) and Debye–Waller parameters (σ, 0.078 Å and 0.039 Å for O and Ni, respectively) were determined from this fit. The filtered R-factor which was 13, and the reduced χ^2^ value was 5.5.

Initially, the reaction was investigated in the presence of two nickel foam electrodes and 99% aldehyde formation was confirmed (Entry 1, Table 2). When the nickel foam electrodes were replaced by platinum electrodes, only 44% aldehyde was obtained (Entry 2). Following these results, other electrodes such as iron foam and graphite produced 2% and 10% products, respectively (Entries 3 and 4). However, the copper electrodes were unstable under the experimental conditions (Entry 5). These experiments revealed that the electrolyte has an essential role in this reaction. Therefore, we examined *n*-Bu_4_NPF_6_, *n-*Bu_4_NBr, *n-*Bu_4_NCl, and LiBr, which gave the product in low to moderate yields (Entries 6–9). A series of solvents that were screened provided unsatisfactory results. (Entries 10–12). We next examined TEMPO and NHS as mediators, but only 15% and 21% yields were obtained, respectively (Entry 13 and 14). To be expected, the absence of NHPI led to a significant drop in the yield (Entry 15). Decreasing NHPI to 10 mol% had minimal harmful effects, and the 1b product was not detected in 5 mA (Entries 16 and 17). The results of the oxidation reaction were the same either in the presence or absence of an external base (Entry 18).

**Table 2:** Optimization of electrochemical oxidation of benzyl alcohol under various conditions (electrodes, solvents, electrolytes, and mediators). For all of these reactions, < 10% maximum error is introduced.


Entry	Anode/cathode	Mediate (X mol%)	Electrolyte	Solvent	Yield (%)^a^
1	Ni foam/ Ni foam	NHPI (20 mol%)	LiClO_4_	CH_3_CN	99
2	Pt/Pt	NHPI (20 mol%)	LiClO_4_	CH_3_CN	44
3	Fe foam/ Fe foam	NHPI (20 mol%)	LiClO_4_	CH_3_CN	2
4	Graphite/graphite	NHPI (20 mol%)	LiClO_4_	CH_3_CN	10
5	Cu foam/Cu foam	NHPI (20 mol%)	LiClO_4_	CH_3_CN	–
6	Ni foam/Ni foam	NHPI (20 mol%)	*n*-Bu_4_NPF_6_	CH_3_CN	13
7	Ni foam/Ni foam	NHPI (20 mol%)	*n*-Bu_4_NBr	CH_3_CN	35
8	Ni foam/Ni Foam	NHPI (20 mol%)	*n*-Bu_4_NCl	CH_3_CN	20
9	Ni foam/Ni foam	NHPI (20 mol%)	LiBr	CH_3_CN	5
10	Ni foam/Ni foam	NHPI (20 mol%)	LiClO_4_	H_2_O(50):CH_3_CN(50)	10
11	Ni foam/Ni foam	NHPI (20 mol%)	LiClO_4_	EtOAc	–
12	Ni foam/Ni foam	NHPI (20 mol%)	LiClO_4_	EtOH	–
13	Ni foam/Ni foam	TEMPO (20 mol%)	LiClO_4_	CH_3_CN	15
14	Ni foam/Ni foam	NHS (20 mol%)	LiClO_4_	CH_3_CN	20
15	Ni foam/Ni foam	-	LiClO_4_	CH_3_CN	50
16	Ni foam/ Ni foam	NHPI (10 mol%)	LiClO_4_	CH_3_CN	80
17^b^	Ni foam/Ni foam	NHPI (20 mol%)	LiClO_4_	CH_3_CN	–
18^c^	Ni foam/ Ni foam	NHPI (20 mol%)	LiClO_4_	CH_3_CN	99

^a^ Product yields were determined by GC analysis.

^b^ I = 5 mA instead of I = 10 mA.

^c^ Adding pyridine as an external base.

Under the optimized concentrations, we then proceeded to employ these conditions for the oxidation of a broad range of alcohols (Table 3). The different electron-donating or electron-withdrawing functional groups of ortho, meta and para situations of benzyl alcohol indicated an excellent conversion and a high selectivity in the corresponding aldehyde (Entries 1–6, Table 3).

**Table 3:** Oxidation of different alcohols using the electrochemical method in mild conditions. For all of these reactions, < 10% maximum error is introduced.


			
**1**, 100% yield, 8:30 h	**2**, 100% yield, 2 h	**3**, 95% yield, 4 h	**4**, 100% yield, 4 h
[Ref]^15^ 95% yield	[Ref]^15^ 72% yield	[Ref]^15^ -	[Ref]^15^ -
[Ref]^26^ 82–99% yield	[Ref]^26^ -	[Ref]^26^ -	[Ref]^26^ -
[Ref]^31^ 66% yield	[Ref]^31^ 83% yield	[Ref]^31^ -	[Ref]^31^ -
[Ref]^37^ 49% yield	[Ref]^37^	[Ref]^37^ -	[Ref]^37^ -
			
**5**, 95% yield	**6**, 100% yield, 4 h	**7**, ND, 8:30 h	**8**, 97%yield, 8:30 h
[Ref]^15^ -	[Ref]^15^ -	[Ref]^15^ -	[Ref]^15^ 71% yield
			
**9**, 90%yield, 8:30 h	**10**, 80%yield, 8:30 h	**11**, 99%yield, 3 h	**12**, 99%yield, 3 h
[Ref]^15^ -	[Ref]^15^ -	[Ref]^15^ -	[Ref]^15^ 75% yield
[Ref]^31^ -	[Ref]^31^ -	[Ref]^31^ -	[Ref]^31^ 78% yield
[Ref]^37^ -	[Ref]^37^ -	[Ref]^37^ 96% yield	[Ref]^37^ 87% yield
			
**13**, 99%yield, 4 h	**14**, 100%yield, 6 h	**15**, 100%yield, 6 h	**16**, ND, 8:30 h
[Ref]^37^ -	[Ref]^37^ 94% yield	[Ref]^37^ -	[Ref]^37^ -
			
**17**, ND, 8:30 h	**18**, 91% yield^b^, 8:30 h		
	Large scale reaction		

Reaction condition: (a) The product yields were determined by GC analysis. (b) Alcohols (10.0 mmol), CH_3_CN (4.9 ml per mmol of alcohol), LiClO_4_ (0.1 M), RT, constant current = 70 mA, 8:30 h.

However, using this procedure for the oxidation of 2, 6-dichlorobenzyl alcohol was not successful (Entry 7, Table 3), which corresponded to the sterically hindered compound. The oxidation of cinnamyl alcohol produced 97% of the cinnamaldehyde (entry 8, Table 3).

The oxidation of heterocyclic alcohols also displayed high yields and strong selectivity (Entries 9, 10, Table 3). Diphenyl alcohol, phenyl ethanol and 1, 2, 3, 4-Tetrahydro-1-naphthol were oxidized to the corresponding ketones with 99% yields (Entries 11, 13, Table 3). The aliphatic alcohols such as cyclohexanol and cycloheptanol could to be oxidized effectively under our experimental conditions (Entries 14, 15, Table 3). However, the anodic oxidation of primary and secondary aliphatic alcohols failed. (Entries 16, 17, Table 3). To investigate the large scale of conversion through this method, oxidation of benzyl alcohol (10.0 mmol) was performed, and 91% aldehyde was obtained (Entry 18, Table 3).

Comparison of the activity of NHPI on nickel foam with other chemical and electrochemical methods in the oxidation of benzyl alcohol is summarized in Table 4.

**Table 4:** Comparison of the efficiency of this method with other existing methods for the oxidation of alcohols.


Conditions	Yields
NaBr (4.0 eq), *N*-hydroxylamines (0.1 eq), CH_2_Cl_2_, NaHCO_3,_ 3.0 *F*/mol, rt^26^	82–99% (A)
NHPI (20 mmol), O_2_ (20 mL/min), CoTPP-Zn_2_Al-LDH (30 mg), CH_3_CN (10 mL), Temperature (80 °C), 24 h^47^	62% (A)
NHPI (10 mol%),CuBr (5 mol%),ethyl acetate (3 mL), O_2_ (0.15 MPa), 75 °C, 20 h^48^	99% (B)
mCPBA (3 mmol), PhI (0.1 mmol), NHPI (0.2 mmol), CH_3_CN:H_2_O (4;1), rt^49^	81% (A)
NHPI (5 mol%),TBN (10 mol%),O_2_-balloon, CH_3_CN, 80 °C^50^	80% (A)
NHPI (10 mol%), HNO_3_ (20 mol%),CuBr_2_ (0.005 mol%), Acetonitrile (9 mL), O_2_ (0.1 MPa), 6 h, 25 °C^51^	50% (A)
NHPI (10 mol%),Co(OAc)_2_ (0.5 mol%),chlorobenzoic acid (5 mol%), Acetonitrile (15 mL), rt, O_2_ at atmospheric pressure^52^	92%
NaNO_3_ (0.83%), HCl (60 ml); chloroform (20 ml) Carbon/stainless steel; temperature (30–34 °C); I = 50 mA^15^	95% (A)
Ni—anode/OH^-^,biphasic system (petroleum ether: H_2_O), 400 cm^2^ nickel, cathode: 400 cm^2^ stell anode, I = 200 mA^31^	66% (A) 3% (B)
NHPI (20 mol%), pyridine (20 mol%),glassy carbon anode, Glassy carbon cathode, NaClO_4_ (0.1 M)^37^	49% (A)
NHPI (20 mol%), LiClO_4_ (0.1 M), CH_3_CN (4.9 mL), undivided cell, Cathode: Ni foam (1 cm^2^)/ anode: Ni foam (1 cm^2^), I = 10 mA, 8:30 h, RT	99% (A)

## Conclusions

In summary, a simple electrochemical method for oxidation of alcohols toward the formation of aldehydes and ketones was developed, using inexpensive, accessible and reusable nickel/nickel oxide surfaces. A variety of alcohols were shown to be compatible with this electrochemical process. The successful oxidation of benzyl alcohol (10.0 mmol) was evaluated. Through the method employed for a larger scale than lab-scale, benzyl alcohol (10 mmol, 1.08 gr) was successfully oxidized to benzaldehyde (91%) without any side or by-products. In the absence of a mediator, nickel/nickel oxide surface produced aldehyde in 50% yield. The nickel/nickel oxide surface was characterized by a number of methods. Especially, X-ray absorption spectroscopy (XAS) showed that after the reaction, the foam had a higher oxidation state than the metallic nickel.

The Fourier transformed EXAFS-spectra of the Ni foam after the reaction showed a high main Ni–O peak that could be simulated by both Ni–O shells at 2.04 Å and 2.45 Å. The spectrum had a Ni–Ni peak corresponding to di-µ-oxo bridged Ni, which could be simulated by three Ni–Ni shells at 2.77 Å, 2.96 Å and 3.11 Å.

## Methods

### Materials

All chemicals were purchased and used without further purification. Lithium perchlorate (LiClO_4_), NHPI, Benzyl alcohol, 4-Methoxybenzyl alcohol, 4-Isopropylbenzyl alcohol, 4-chlorobenzyl alcohol, 3-chlorobenzyl alcohol, 2,4-dichlorobenzyl alcohol, 2,6-dichlorobenzyl alcohol, cinnamyl alcohol, 3-pyridyl methanol, furfuryl alcohol, diphenylmethanol, phenyl ethanol, 1,2,3,4-tetrahydro-1-naphthol, cyclohexanol, cycloheptanol, octanol and 2-nonanol were purchased from Merck Company.

The nickel foam (Nanobazar), copper foam (Nanobazar), Fe foil (Suzhou JSD Foam Metal Co., Ltd), and graphite (Nanobazar) were purchased from commercial sources. All reactions were performed under atmospheric oxygen or air in test tube flasks. The reactions were followed by Thin-layer chromatography (TLC) on silica gel plates and Gas chromatography (GC). Preparative thin-layer chromatography (PTLC) separations were carried out on 0.25 or 0.5 mm E. Merck silica gel plates (60F-254). The following abbreviations were used to explain the multiplicities: s = singlet, d = doublet, t = triplet, q = quartet, m = multiple.

### Preparation of the electrodes

All of the reactions were carried out in an experimental setup. The different electrodes used in this reaction are shown in Fig. S1.

For each measurement, a 1.0 cm × 1.0 cm piece of foam nickel (10 cm × 10 cm) with a thickness of1.5 mm was cut. A piece of glass was placed between the two electrodes which were then connected with parafilm. The bottom half of the electrodes were immersed to the solution, to avoid collision with the stirring bar. A septum was chosen, above which two small incisions were made. Steel wire was threaded through these incisions and attached to each of the electrodes. The assembled electrodes were used as described (Fig. S2).

### Electrochemical oxidation of benzyl alcohols^46^

The test tube and all measuring instruments were carefully washed with acetone. No attempt was made to remove oxygen or water at any stage. Lithium perchlorate (0.6 mmol, 64.0 mg) and NHPI (0.2 mmol, 32.0 mg) were dissolved in 5.0 mL acetonitrile, and the mixture was stirred for 10 min to form a homogeneous solution. Alcohol (1.0 mmol) was added to the reaction mixture, which was mixed until the solution was homogeneous. The pre-prepared electrodes were partially immersed to the solution. Parafilm was wrapped around the septum entirely to keep the solvent constant. After the connection was complete, a constant voltage (4.0 V) was applied and the current was measured, which showed a relatively constant current of 10 mA. After completion of the reaction, the electrodes were washed with EtOAc (10 mL) and, the combined organic phase was washed with H_2_O (2 × 5 mL) and brine (1 × 5 ml). The organic phase was dried over anhydrous MgSO_4_. The solvent was evaporated and purified by flash column with hexane–EtOAc (9:1) to give a corresponding aldehyde or ketone.

## Supplementary information

Supplementary Information.
