# Supplementary material for: Electrochemical alcohols oxidation mediated by N-hydroxyphthalimide on nickel foam surface
Source: Sci Rep. 2020 Nov 9;10:19378. doi: 10.1038/s41598-020-75397-8 (PMC7653038; doi:10.1038/s41598-020-75397-8)
Supplement: Supplementary file 1 — Supplementary Information. [file 41598_2020_75397_MOESM1_ESM.docx]

**Supporting Information**

**Electrochemical alcohol oxidation mediated by *N*-hydroxyphthalimide on nickel foam surface**

Leila Behrouzi,^a^ Robabeh Bagheri,^b^ Mohammad Reza Mohammadi,^c^ Zhenlun Song,^d^ Petko Chernev,^e,f^ Holger Dau,^f^ Mohammad Mahdi Najafpour*^a,g,h^ and Babak Kaboudin*^a^

^a^Department of Chemistry, Institute for Advanced Studies in Basic Sciences (IASBS), Zanjan, 45137-66731, Iran

^b^School of Physical Science and Technology, College of Energy, Soochow Institute for Energy and Materials Innovations and Key Laboratory of Advanced Carbon Materials and Wearable Energy Technologies of Jiangsu Province, Soochow University, Suzhou 215006, China

^c^University of Sistan and Baluchestan, Department of Physics, Zahedan, Iran, 98167-45845.

^d^Surface Protection Research Group, Surface Department, Ningbo Institute of Materials Technology and Engineering, Chinese Academy of Sciences, 519 Zhuangshi Road, Ningbo 315201, China

^e^Uppsala University, Department of Chemistry - Ångströmlaboratoriet, Lägerhyddsvägen 1, 75120 Uppsala, Sweden

^f^Freie Universität Berlin, Fachbereich Physik, Arnimallee 14, 14195 Berlin, Germany

^g^Center of Climate Change and Global Warming, Institute for Advanced Studies in Basic Sciences (IASBS), Zanjan, 45137-66731, Iran

^h^Research Center for Basic Sciences & Modern Technologies (RBST), Institute for Advanced Studies in Basic Sciences (IASBS), Zanjan 45137-66731, Iran

*Corresponding authors: Tel: (+98) 24 3315 3220; E-mail: kaboudin@iasbs.ac.ir (B.K.); Tel: (+98) 24 3315 3201; E-mail: mmnajafpour@iasbs.ac.ir (M.M.N.)*

**Table of Contents**

| 1. Instrument | 3 |
| --- | --- |
| 2. Figures and Pictures | 4,5 |
| 2. 13C NMR and 1H NMR of products | 6,7 |
| 3. 13C NMR and 1H NMR spectra of products | 8- |
| ^13^C NMR and ^1^H NMR spectrum of benzaldehyde | 8 |
| ^13^C NMR and ^1^H NMR spectrum of 4-chlorobenzaldehyde | 9 |
| ^13^C NMR and ^1^H NMR spectrum of 4-methoxybenzaldehyde | 10 |
| ^13^C NMR and ^1^H NMR spectrum of 4-isopropylbenzaldehyde | 11 |
| ^13^C NMR and ^1^H NMR spectrum of cinnamaldehyde | 12 |
| ^13^C NMR and ^1^H NMR spectrum of 3-chlorobenzaldehyd | 13 |
| ^13^C NMR and ^1^H NMR spectrum of furan-2-carbaldehyde | 14 |
| ^13^C NMR and ^1^H NMR spectrum of cyclohexanone | 15 |
| ^13^C NMR and ^1^H NMR spectrum of cycloheptanone | 16 |
| ^13^C NMR and ^1^H NMR spectrum of acetophenone | 17 |
| ^13^C NMR and ^1^H NMR spectrum of benzophenone | 18 |
| ^13^C NMR and ^1^H NMR spectrum of 1-Tetralone | 19 |
| References | 20 |

**Instrumentation**

Scanning electron microscopy (SEM) was carried out with VEGA\\TESCAN-XMU. X-ray powder diffraction (XRD) patterns were recorded with a Bruker D8 ADVANCE (Germany) diffractometer (CuK_α_ radiation). Electrochemical experiments were performed using an EmStat3+ from PalmSens (Netherlands). The distance between two opposite sides of the electrode was measured by a digital caliper MarCal 16ER model (Mahr, Germany) and was used to calculate the geometric current density (j). The temperature was measured by a Laser liner 082 (Germany). FTIR spectra of KBr pellets of compounds were recorded on a Bruker vector 22 in the range between 400 and 4000 cm^−1^. UV-Visible spectroscopy was carried out with Pharmacia biotech ultrospec 3100. The high resolution in situ visible spectra were recorded by a mini spectrophotometer (Pooyesh Tadbir Karaneh (Phystec), Iran).

XAS measurements (EXAFS, XANES) at the nickel K-edges for the separated powder on the surface of the electrode were performed at the KMC-3 beamline at the BESSY II synchrotron facility (Helmholtz-Zentrum Berlin, Germany). The measurements were performed at 20 K using a liquid-helium-cooled cryostat (Oxford-Danfysik) in the top-up mode of the BESSY II storage ring at 250 mA ring current. The angle between the sample surface and the incoming x-ray beam was ~45°. The fluorescence-detected X-ray absorption spectra at the K-edge were collected using a 13–element Ge detector (Ultra-LEGe detector, Canberra GmbH) installed perpendicular to the incident X-ray beam.

**Large-scale benzyl alcohol oxidation^[1]^**

The method was similar to previously reported method:^[1]^

Throughout this process, no attempt was made to exclude air. NHPI (1.96mmol, 320.0 mg), LiClO_4_ (6.0 mmol, 640.0 mg) and acetonitrile (49.0 ml) were added to a beaker, and the resulting suspension was stirred until the majority of the solids had dissolved. Benzyl alcohol (1.0 ml, 10.0 mmol) was then added to the solution. The electrodes (10.0 cm ×10.0 cm nickel foam) were partially submerged into the solution, and the beaker was sealed with parafilm to minimize solvent evaporation (Fi. S3). The reaction mixture was electrolyzed at a current of 70 mA until the reaction was completed as assessed by TLC analysis. After completion of the reaction, the electrodes were washed with EtOAc (30 mL), and all combined organic phase was washed with H2O (2× 30 mL) and brine (1× 30 ml). The organic phase dried over anhydrous MgSO_4_. The solvent was evaporated and was purified by flash column with hexane-EtOAc (9:1) to give benzaldehyde at a 91% yield.

**Cyclic voltammetry for benzyl alcohol in the presence and absence of NHPI**

Cyclic voltammetry studies were performed out with a three-electrode setup in which Ni foam, Ag|AgCl|KClsat and Ni foam served as the working, reference, and auxiliary electrodes, respectively. The experiments were undertaken at room temperature using a 100 mV scan rate, with Lithium perchlorate used as the electrolyte (120 mM), NHPI (40 mM) and benzyl alcohol (200 mM). Electrochemical experiments were performed using an EmStat3+device from PalmSens (Netherlands).

**1. Figures and pictures**


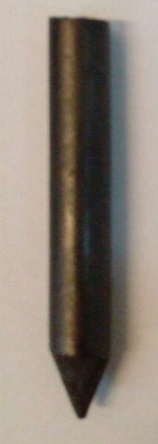

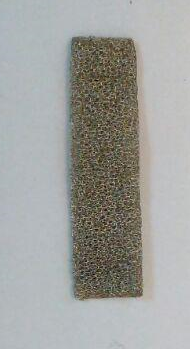

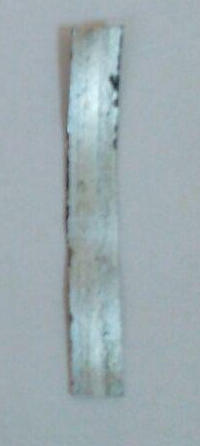

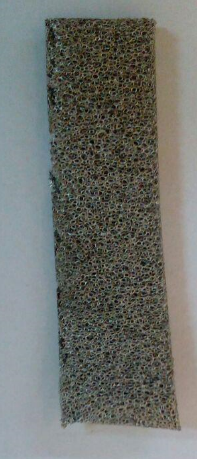

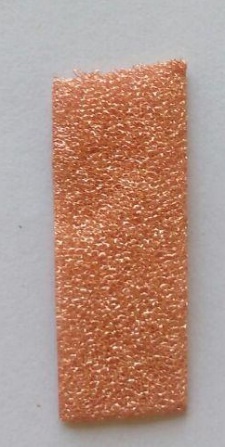


**Fig. S1** Graphite, a nickel foam, a platinum foil, an iron foam, a copper foam (left to right).

| 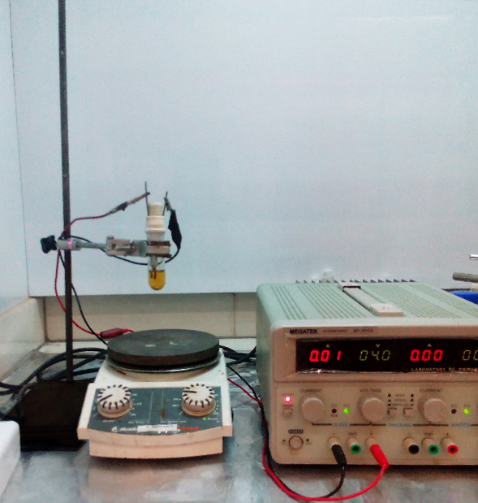 | 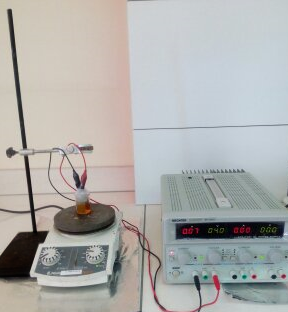 |
| --- | --- |
| **Fig S2** Set-up for the reaction. | **Fig. S3** Set-up for a large-scale oxidation |


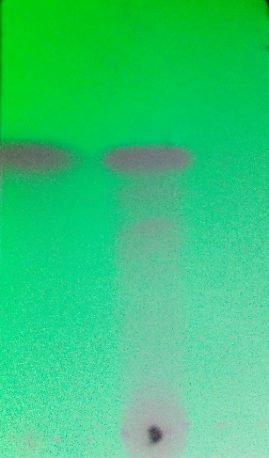


**Fig. S4** The spot of aldehyde (left) and the spot of mixed reaction (right).


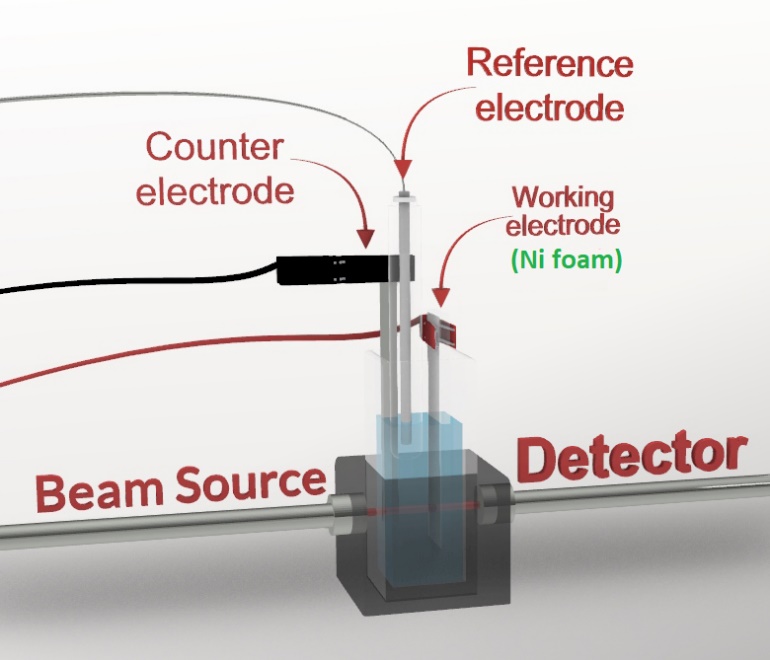


**Fig. S5** Set-up for spectroelectrochemistry.

**Fig. S6** k^3^-weighted χ(k) of Ni foam after the reaction at 1.4 V for 2 h in phosphate buffer (1.0 M, pH = 6.7). The blue and red lines show the experimental data and simulation, respectively. Phase shift not corrected. The fit parameters for the simulations are given in Table 2.

**2. ^13^C NMR and ^1^H NMR of the products:**

| **Benzaldehyde**   | The characterization data obtained for benzaldehyde were identical to those previously reported in the literature^[^[^2^](#_ENREF_3)^]^. ^1^H NMR (400 MHz, CDCl_3_): δ 10.06 (s, 1H), 7.92 (d, J = 8.0 Hz, 2H), 7.70 – 7.64 (m, 1H), 7.57 (t, J = 7.5 Hz, 2H). ^13^C NMR (101 MHz, CDCl_3_): δ 192.40, 136.44, 134.48, 129.76, 129.02. |
| --- | --- |
| **4-Chlorobenzaldehyde**   | The characterization data obtained for 4-chlorobenzaldehyde were identical to those previously reported in the literature^[^[^3^](#_ENREF_5)^]. 1^H NMR (400 MHz, CDCl_3_): δ 9.98 (s, 1H), 7.81 (d, J = 8.5 Hz, 2H), 7.50 (d, J = 8.4 Hz, 2H). ^13^C NMR (101 MHz, CDCl_3_): δ 190.94, 140.99, 134.71, 130.95, 129.49. |
| **4-Methoxybenzaldehyde**   | The characterization data obtained for 4-methoxybenzaldehyde were identical to those previously reported in the literature^[^[^3^](#_ENREF_5)^]^. ^1^H NMR (400 MHz, CDCl_3_): δ 9.78 (s, 1H), 7.73 (d, J = 8.4 Hz, 2H), 6.90 (d, J = 8.4 Hz, 2H), 3.77 (s, 3H). ^13^C NMR (101 MHz, CDCl_3_): δ 190.69, 164.54, 131.86, 129.87, 114.25, 55.47. |
| **4-Iso-propylbenzaldehyde**   | The characterization data obtained for 4-Iso-propylbenzaldehyde were identical to those previously reported in the literature^[^[^4^](#_ENREF_6)^]^. ^1^H NMR (300 MHz, CDCl_3_): δ = 9.98 (s, 1 H), 7.82 (d, J = 8.1 Hz, 2 H), 7.40 (d, J =8.1 Hz, 2 H), 3.00 (h, J = 6.9 Hz, 1 H), 1.29 (d, J = 6.9 Hz, 6 H). 13C NMR (75 MHz, CDCl3): δ = 191.99, 156.18, 134.48, 129.94, 127.08, 34.41, 29.63, 23.56. |
| **Cinnamaldehyde**   | The characterization data obtained for cinnamaldehyde were identical to those previously reported in the literature^[^[^2b^](#_ENREF_4)^]^. ^1^H NMR (400 MHz, CDCl_3_): δ 9.75 (dd, J = 7.7, 1.0 Hz, 1H), 7.61 (dd, J = 5.6, 2.1 Hz, 2H), 7.54 (s, 1H), 7.50 (s, 1H), 7.46 (d, J = 9.2 Hz, 2H), 6.76 (ddd, J = 16.0, 7.7, 0.9 Hz, 1H). ^13^C NMR (101 MHz, CDCl_3_): δ 193.76, 152.83, 134.04, 131.31, 129.14, 128.64, 128.53. |
| **3-Chlorobenzaldehyde**    **furan-2-carbaldehyde**   | The characterization data obtained for 3-Chlorobenzaldehyde were identical to those previously reported in the literature^[^[^5^](#_ENREF_7)^]^. 1H NMR (400MHz, CDCl_3_) d 7.46 (d, J = 6.5 Hz, 1H), 7.54 (d, J = 7.0 Hz, 1H), 7.73 (d, J = 6.5 Hz, 1H), 7.78 (s, 1H), 9.94 (s, 1H); 13C NMR (101MHz, CDCl_3_) δ 190.7, 137.7, 135.3, 134.2, 130.3, 129.1, 127.9.  The characterization data obtained for furan-2-carbaldehyde were identical to those previously reported in the literature^[^[^5^](#_ENREF_8)^]^. ^1^H NMR (400 MHz, CDCl_3_): δ 6.58 (t, 1H), 7.23 (dd, 1H), 7.24 (dd, 1H), 9.6 (s, 1H) ppm; ^13^C NMR (101 MHz, CDCl_3_): δ179.7, 153, 148, 120, 110ppm. |
| **Cyclohexanone**   | The characterization data obtained for cyclohexanone were identical to those previously reported in the literature. ^1^H NMR (400 MHz, CDCl_3_): δ 2.33 (t, J = 6.4 Hz, 2H), 1.90 – 1.82 (m, 2H), 1.76 – 1.67 (m, 1H).^13^C NMR (101 MHz, CDCl_3_): δ 212.08, 41.96, 27.01, 24.98. |
| **Cycloheptanone**   | The characterization data obtained for cycloheptanone were identical to those previously reported in the literature. ^1^H NMR (400 MHz, CDCl_3_): δ 1.67 (m, 4H), 1.72 (m, 4H), 2.5 (t, 4H).^13^C NMR (101 MHz, CDCl_3_): δ 214, 43, 30, and 22. |
| **Acetophenone**    **Benzophenone**    [**1-Tetralone**](https://www.google.com/url?sa=t&rct=j&q=&esrc=s&source=web&cd=2&cad=rja&uact=8&ved=2ahUKEwjr17-9ofTcAhVCzRoKHUGzDnUQFjABegQIBhAB&url=https%3A%2F%2Fen.wikipedia.org%2Fwiki%2F1-Tetralone&usg=AOvVaw2y2_B2iYXr0XsZ9oSNJesp)   | The characterization data obtained for acetophenone were identical to those previously reported in the literature^[^[^7^](#_ENREF_9)^]^. ^1^H NMR (400 MHz, CDCl_3_): δ 7.97 (d, J = 8.0 Hz, 2H), 7.58 (t, 1H), 7.47 (t, 2H), 2.61 (s, 3H). ^13^C NMR (101 MHz, CDCl_3_): δ 198.14, 137.14, 133.11, 128.58, 128.31, 26.0.  The characterization data obtained for benzophenone were identical to those previously reported in the literature^[^[^8^](#_ENREF_10)^]^. ^1^H NMR (400 MHz, CDCl_3_): δ 7.49 (t, J = 7.2 Hz, 4H), 7.59 (d, J = 7.2 Hz, 2H), 7.81 (d, J = 7.2 Hz, 4H) ppm; ^13^C NMR (101 MHz, CDCl_3_): δ 128.3, 130.1, 132.4, 137.6, 196.7 ppm.  The characterization data obtained for benzophenone were identical to those previously reported in the literature^[^[^9^](#_ENREF_11)^]^. ^1^H NMR (400 MHz, CDCl_3_) δ 7.94 (d, J = 8.0 Hz, 1H), 7.37 (dt, J = 7.6, 1.2 Hz, 1H), 7.20 (t, J = 7.2 Hz, 1H), 7.15 (d, J = 7.6 Hz, 1H), 2.87 (t, J = 6.0 Hz, 2H), 2.56 (t, J = 6.4 Hz, 2H), 2.08-2.00 (m, 2H); ^13^C NMR (101 MHz, CDCl_3_) δ 198.4, 144.5, 133.4, 132.6, 128.8, 127.1, 126.6, 39.2, 29.7, 23.3  . |

**7. ^13^C NMR and ^1^H NMR spectra of products.**

**^13^C NMR spectrum of benzaldehyde:**

**
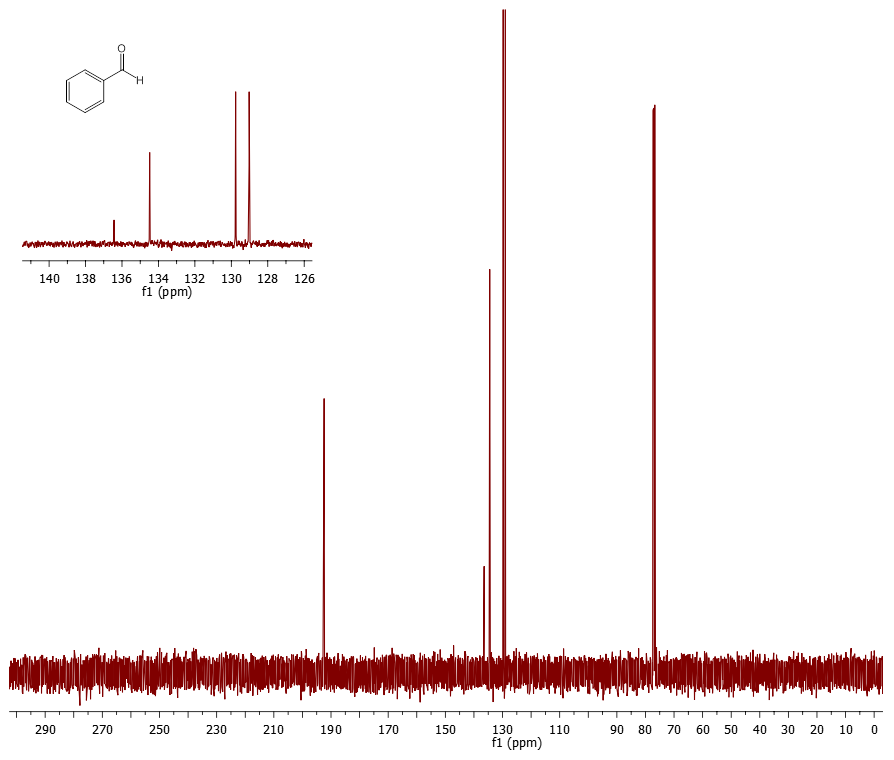
**

**^1^H NMR spectrum of benzaldehyde:**


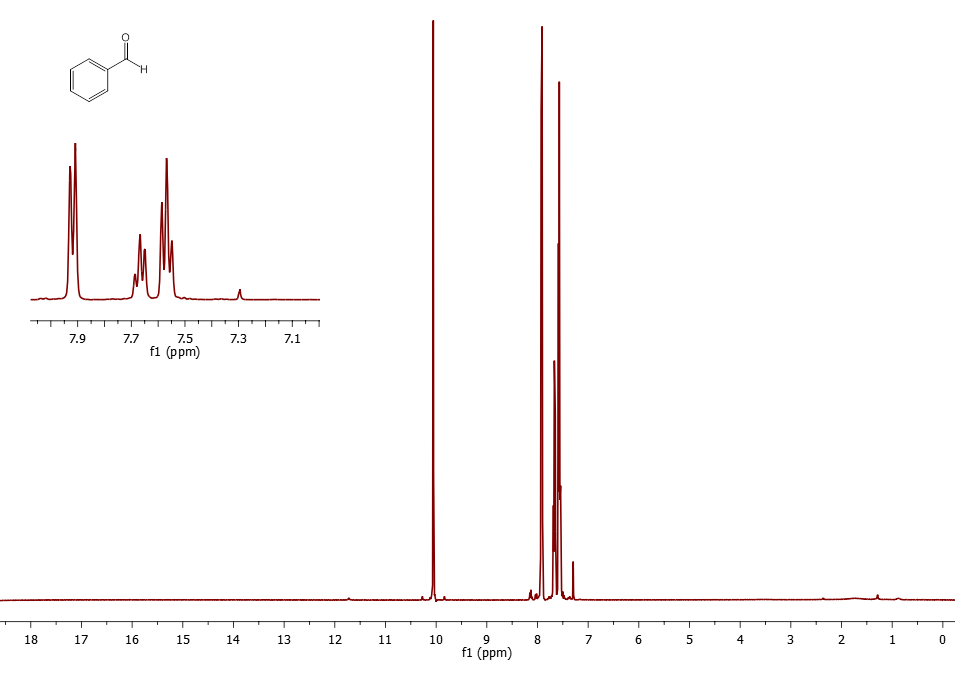


**^13^C NMR spectrum of 4-chlorobenzaldehyde:**

**
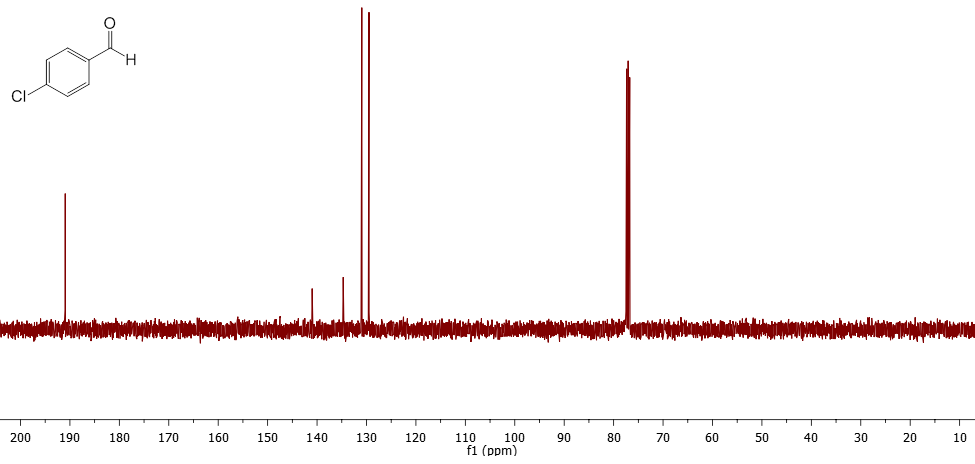
**

**^1^H NMR spectrum of 4-chlorobenzaldehyde:**

**
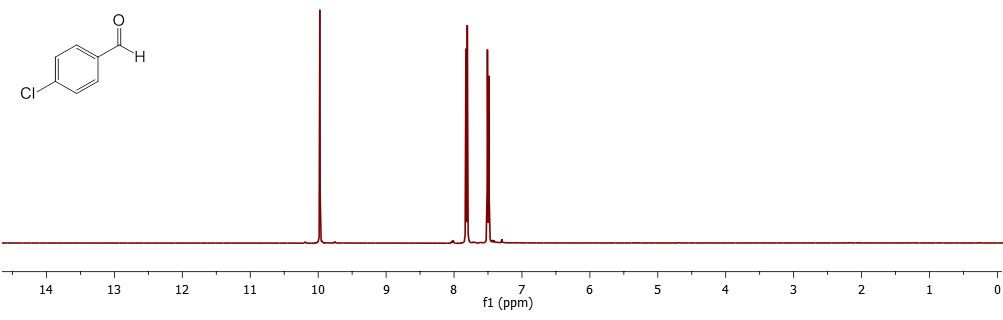
**

**^13^C NMR spectrum of 4-methoxybenzaldehyde:**

**
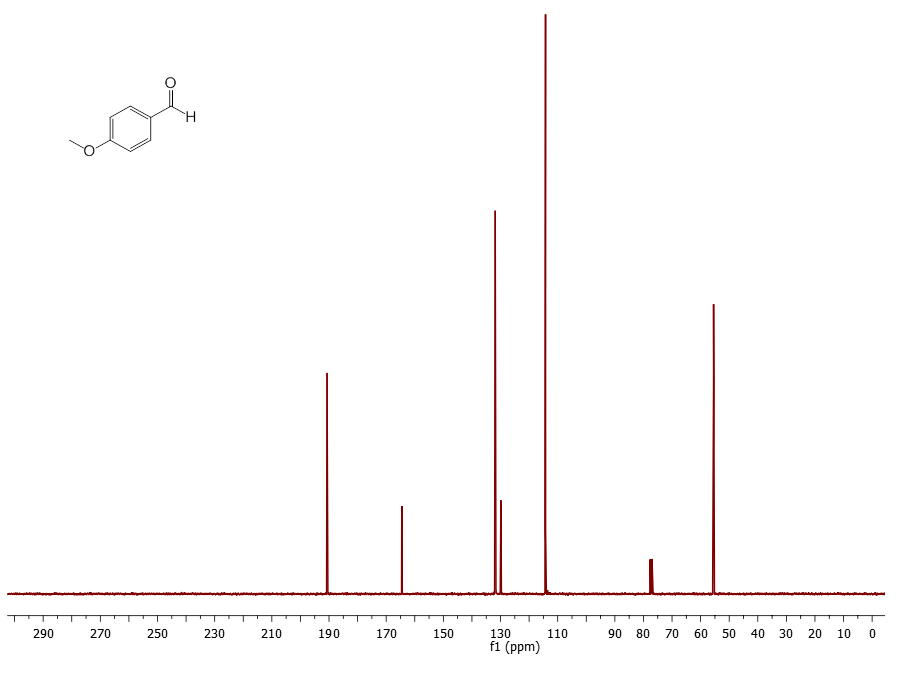
**

**^1^H NMR spectrum of 4-methoxybenzaldehyde:**


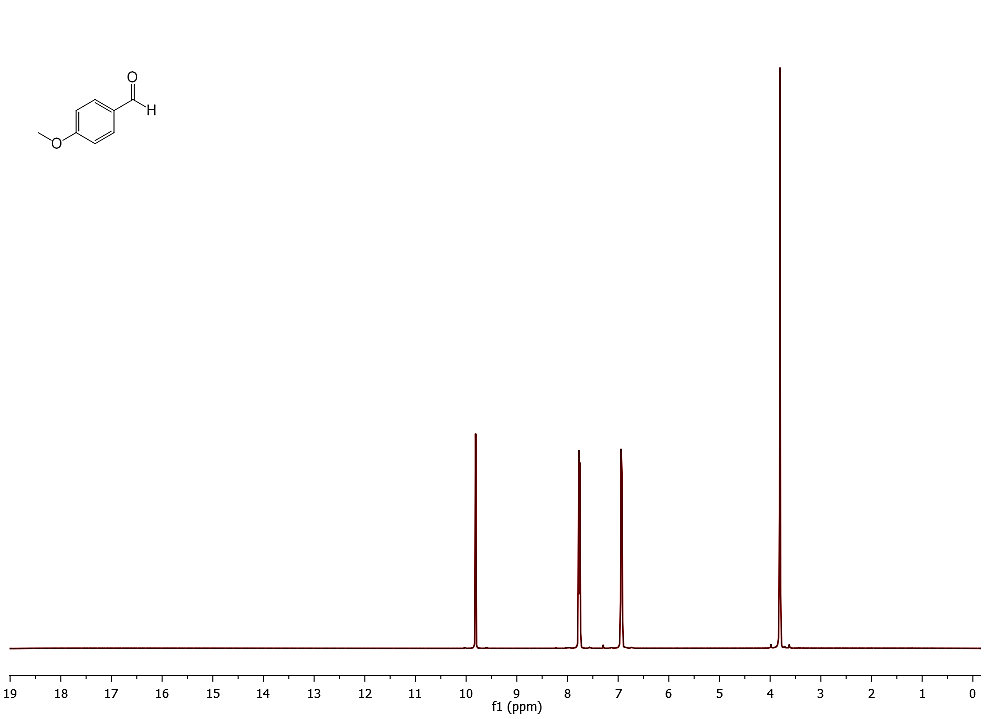


**^1^H NMR spectrum of 4-isopropylbenzaldehyde:**

**
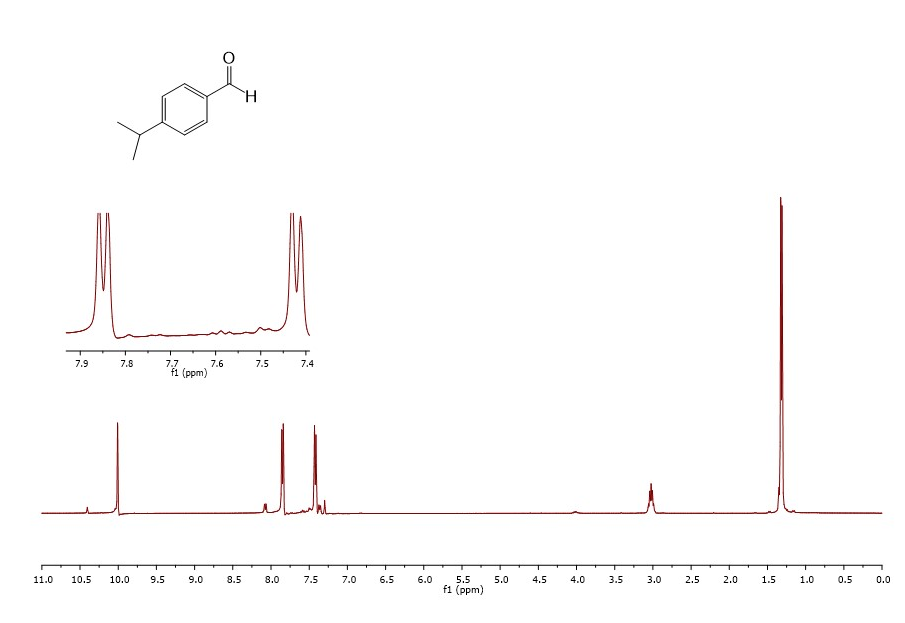
**

**^13^C NMR spectrum of 4-isopropylbenzaldehyde:**

**
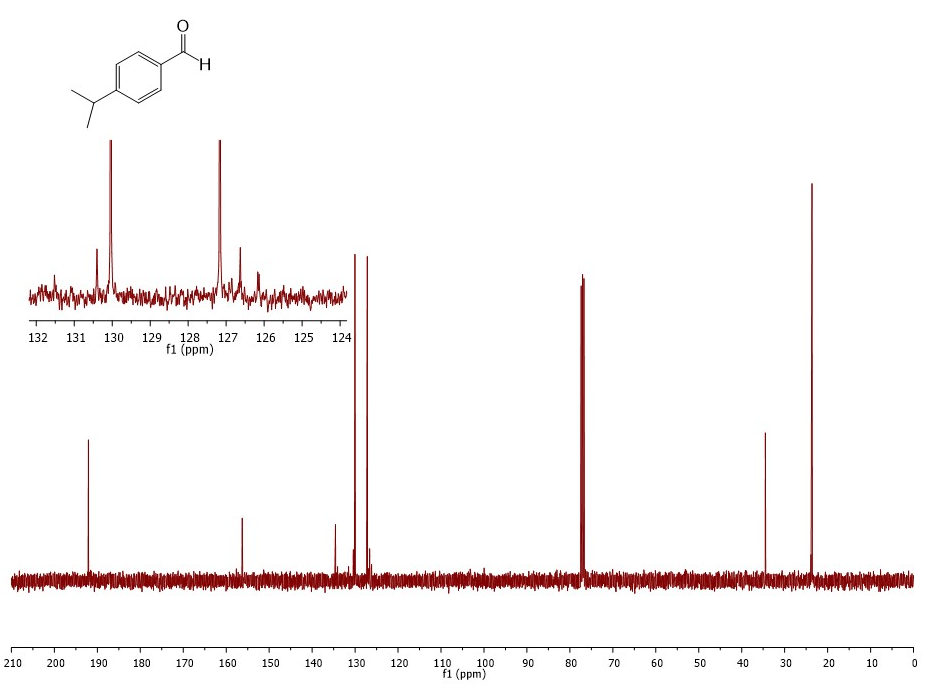
**

**^13^C NMR spectrum of cinnamaldehyde:**

**
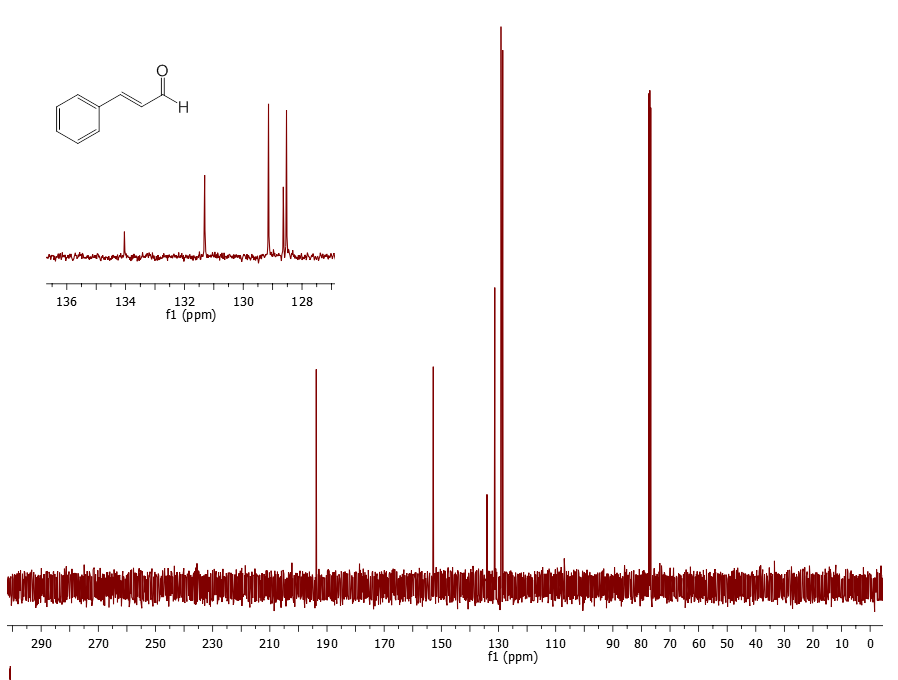
**

**^1^H NMR spectrum of cinnamaldehyde:**


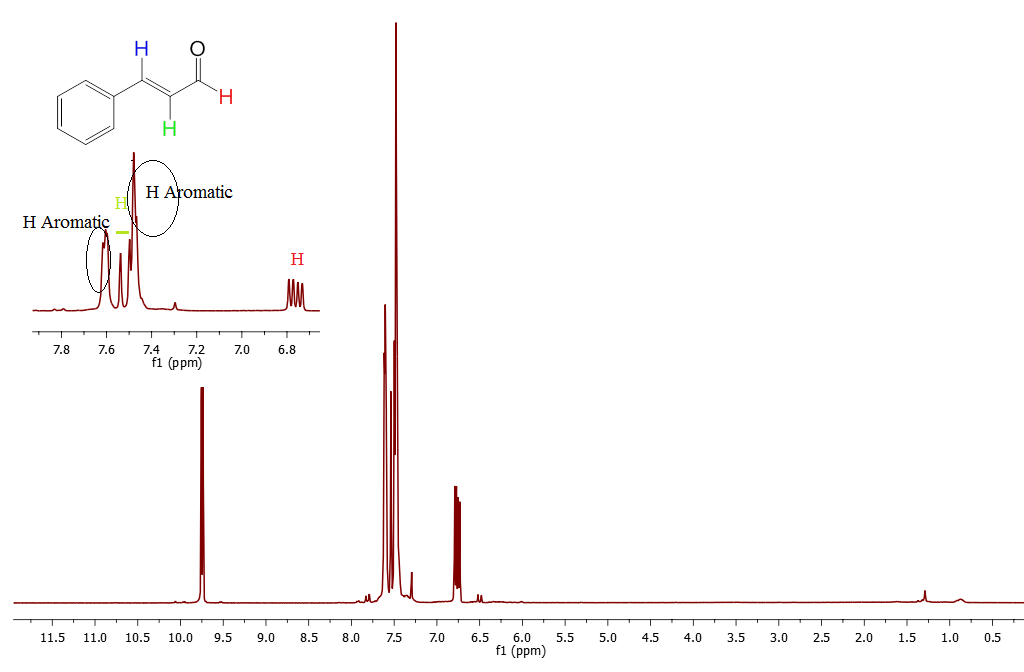


**^13^C NMR spectrum of 3-chlorobenzaldehyde:**


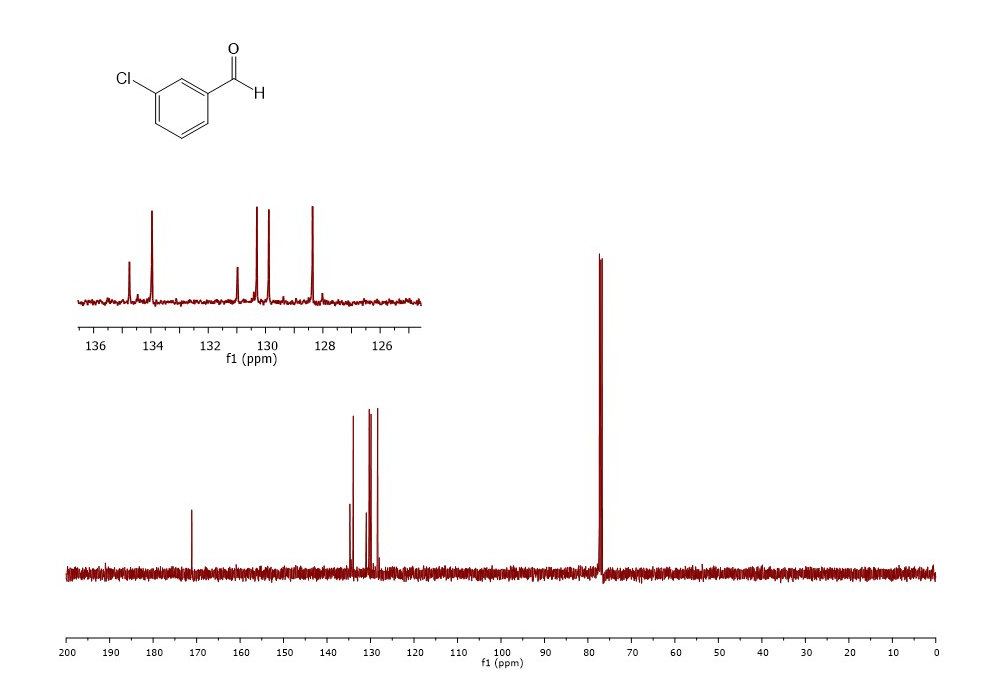


**^1^H NMR spectrum of 3-chlorobenzaldehyde:**


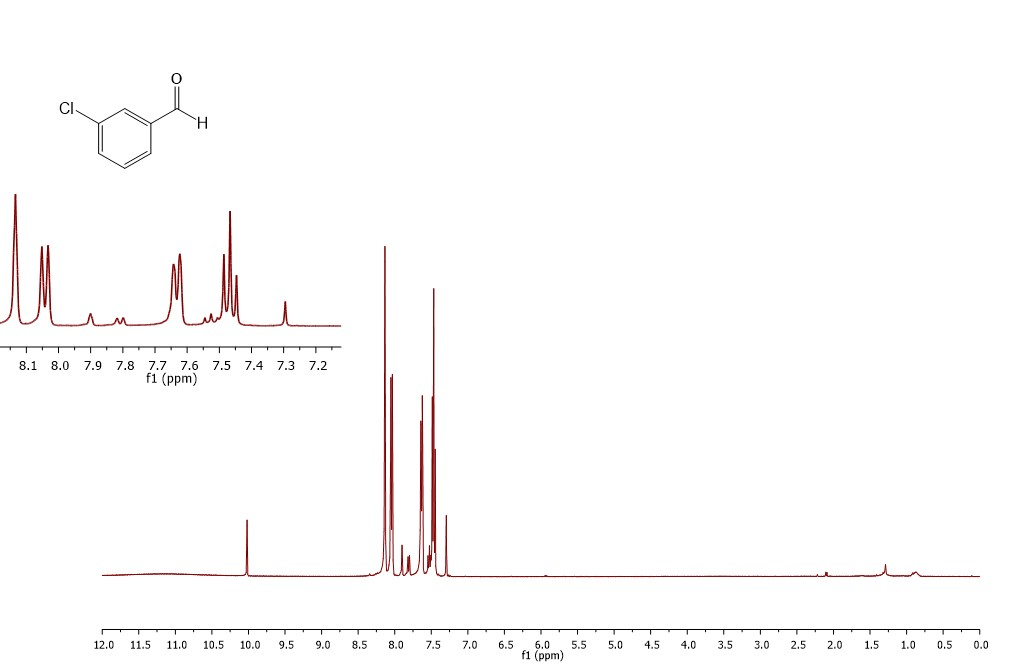


**^13^C NMR spectrum of furan-2-carbaldehyde:**

**
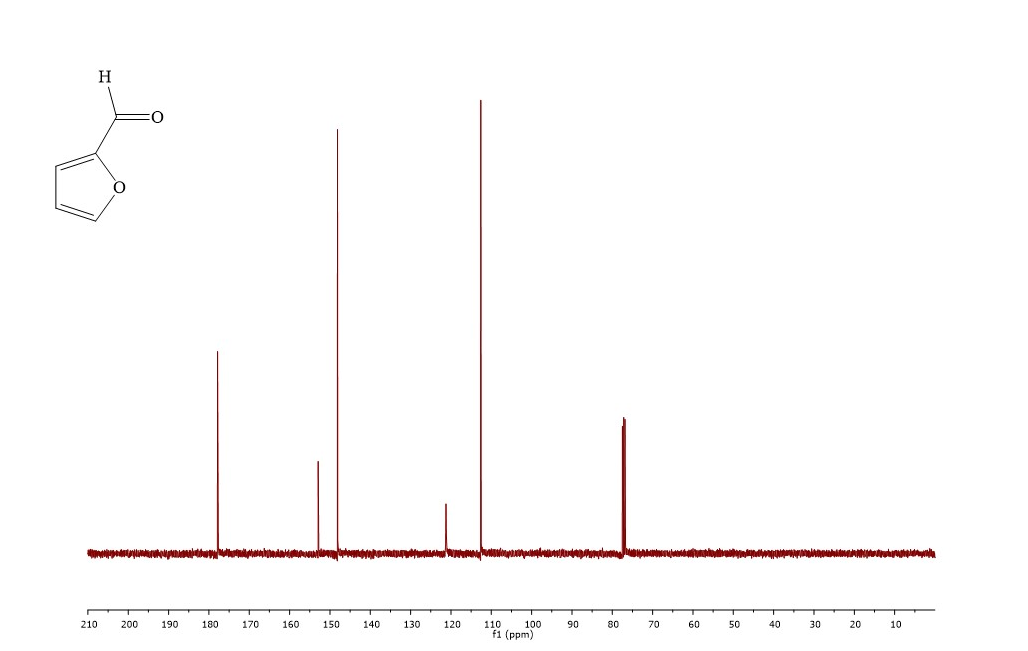
**

**^1^H NMR spectrum of furan-2-carbaldehyde:**


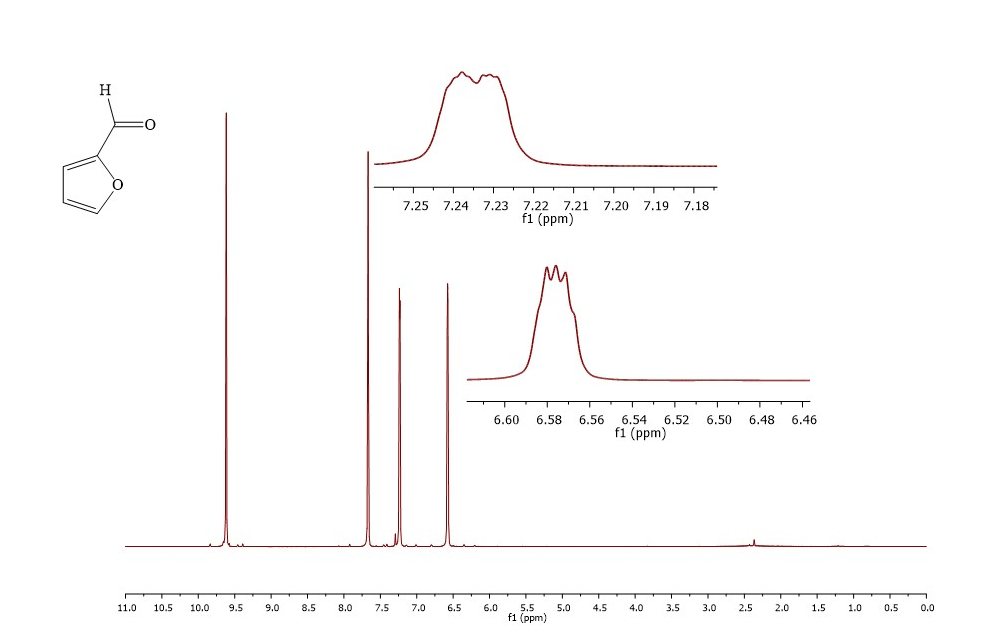


**^13^C NMR spectrum of cyclohexanone:**


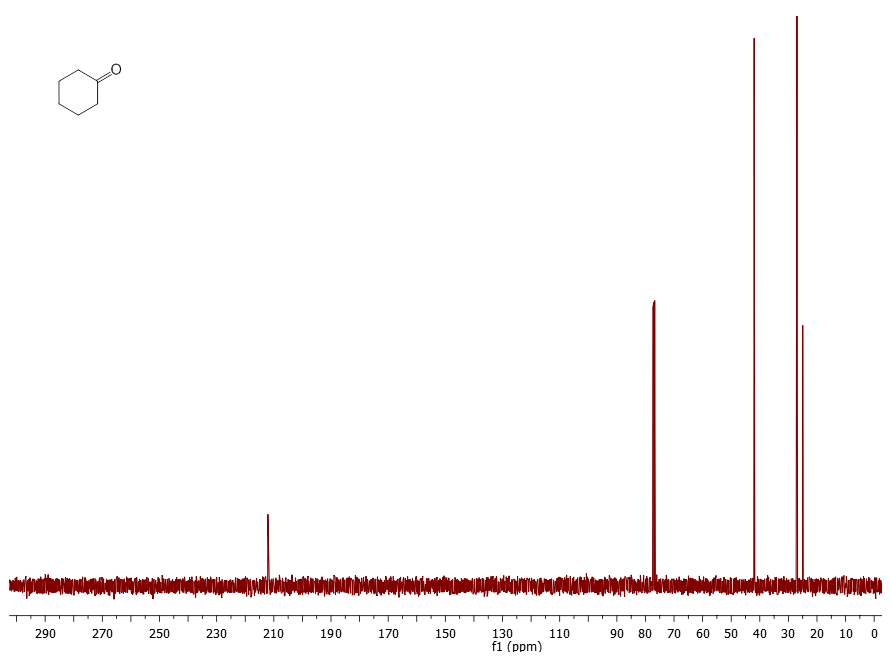


**^1^H NMR spectrum of cyclohexanone:**


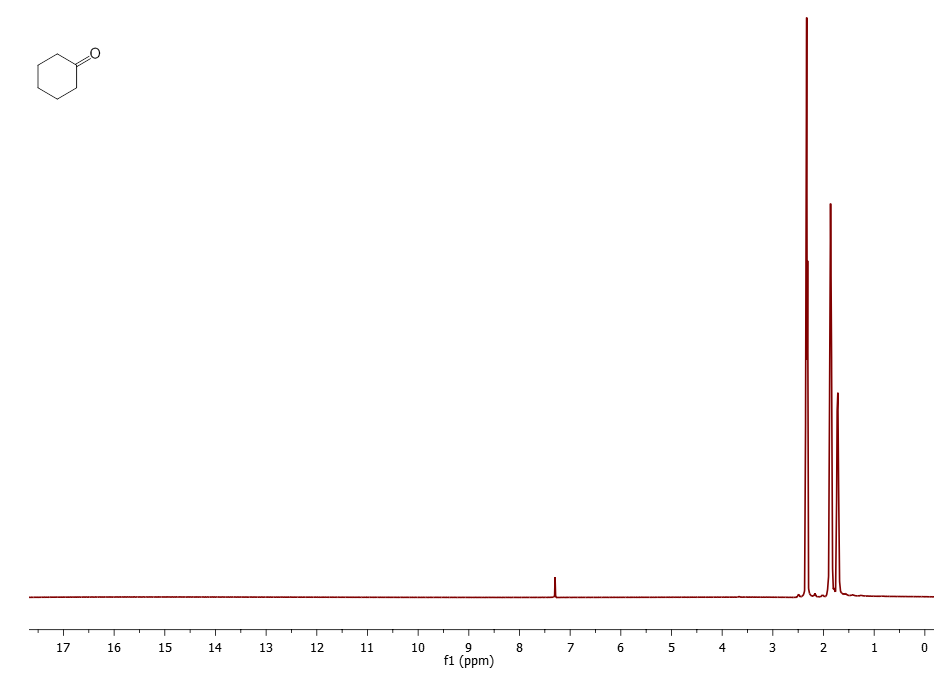


**^13^C NMR spectrum of cycloheptanon:**

**
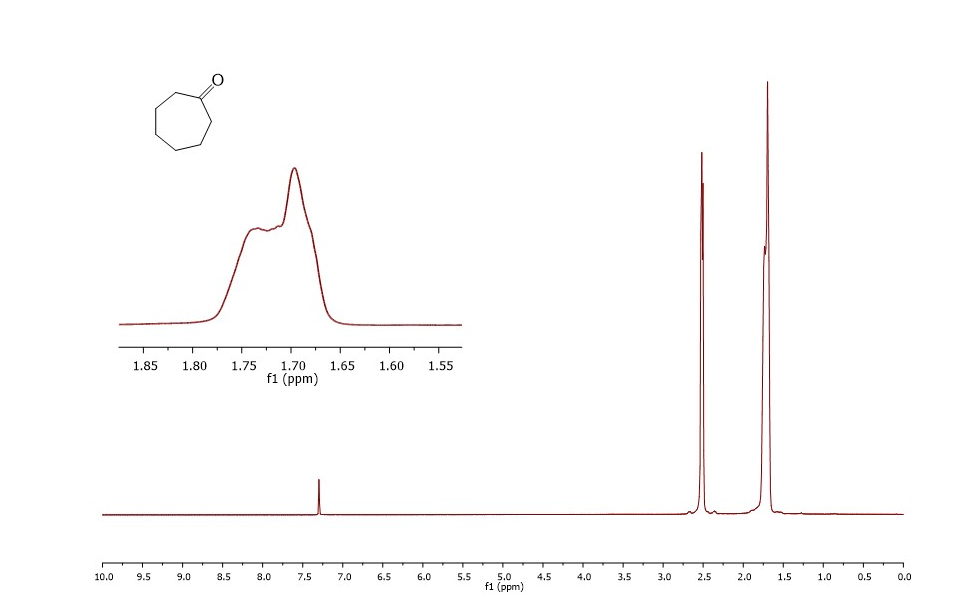
**

**^13^H NMR spectrum of Cycloheptanon:**

**
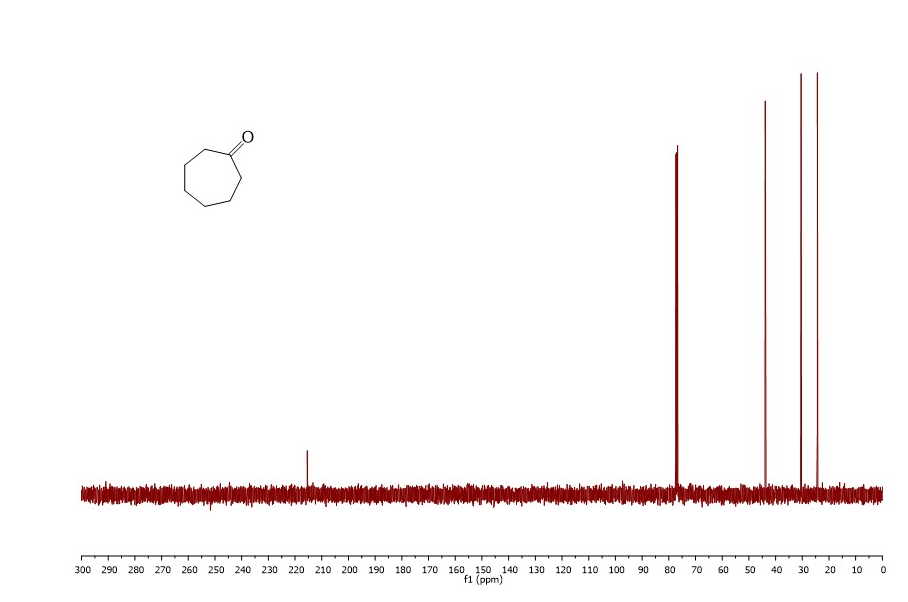
**

**^13^C NMR spectrum of acetophenone:**


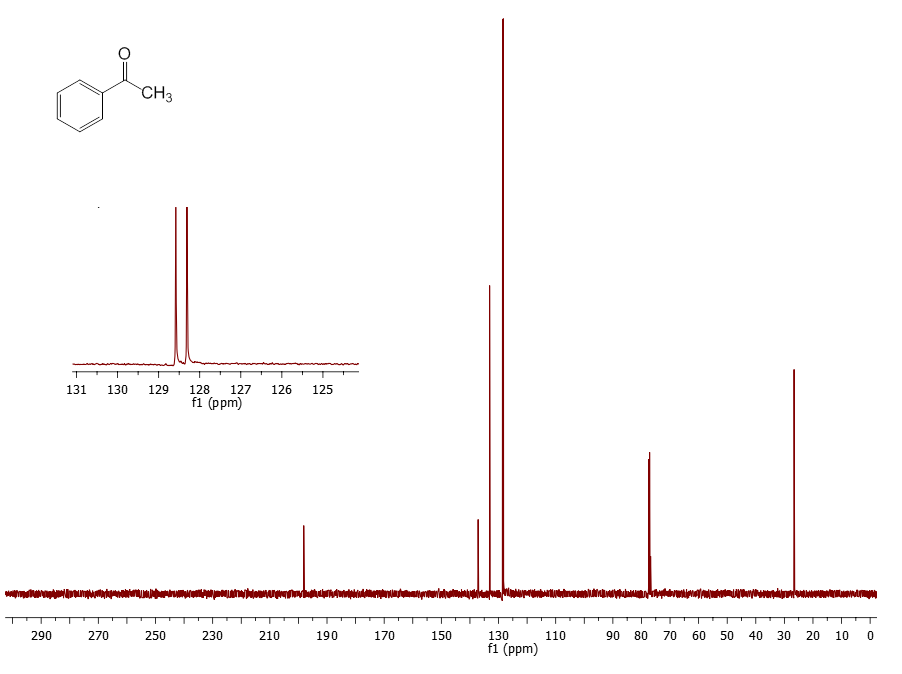


**^13^H NMR spectrum of acetophenone:**


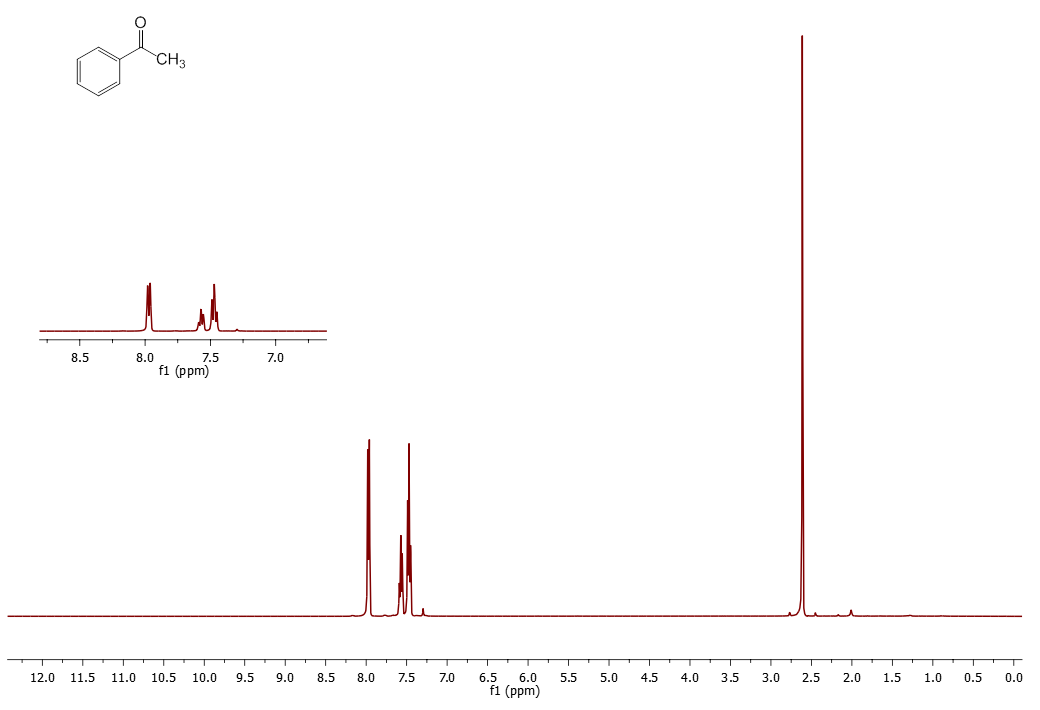


**^13^C NMR spectrum of benzophenone:**

**
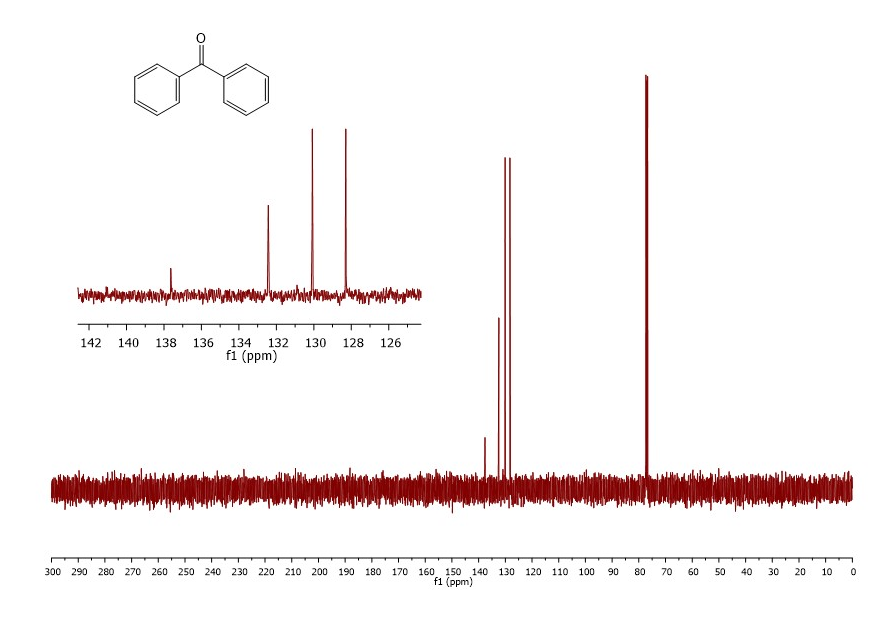
**

**^1^H NMR spectrum of benzophenone:**

**
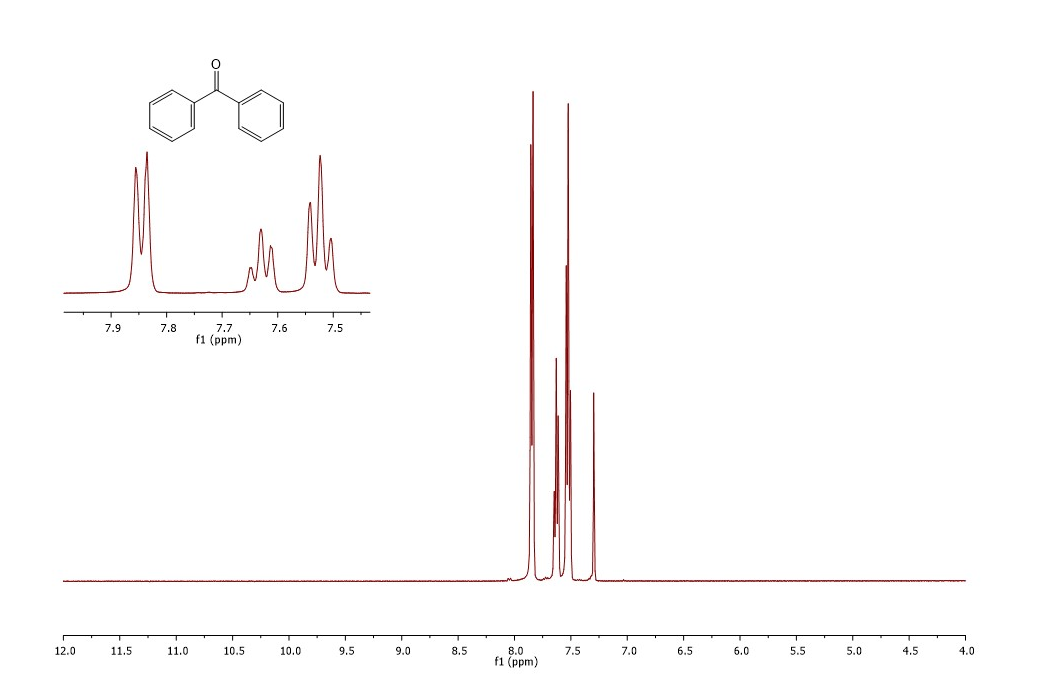
**

**^13^C NMR spectrum of** [**1-tetralone**](https://www.google.com/url?sa=t&rct=j&q=&esrc=s&source=web&cd=5&cad=rja&uact=8&ved=2ahUKEwjr17-9ofTcAhVCzRoKHUGzDnUQFjAEegQIARAB&url=https%3A%2F%2Fpubchem.ncbi.nlm.nih.gov%2Fcompound%2Falpha-Tetralone&usg=AOvVaw3-dUlKOTWyjewjSqsq4bUv)**:**

**
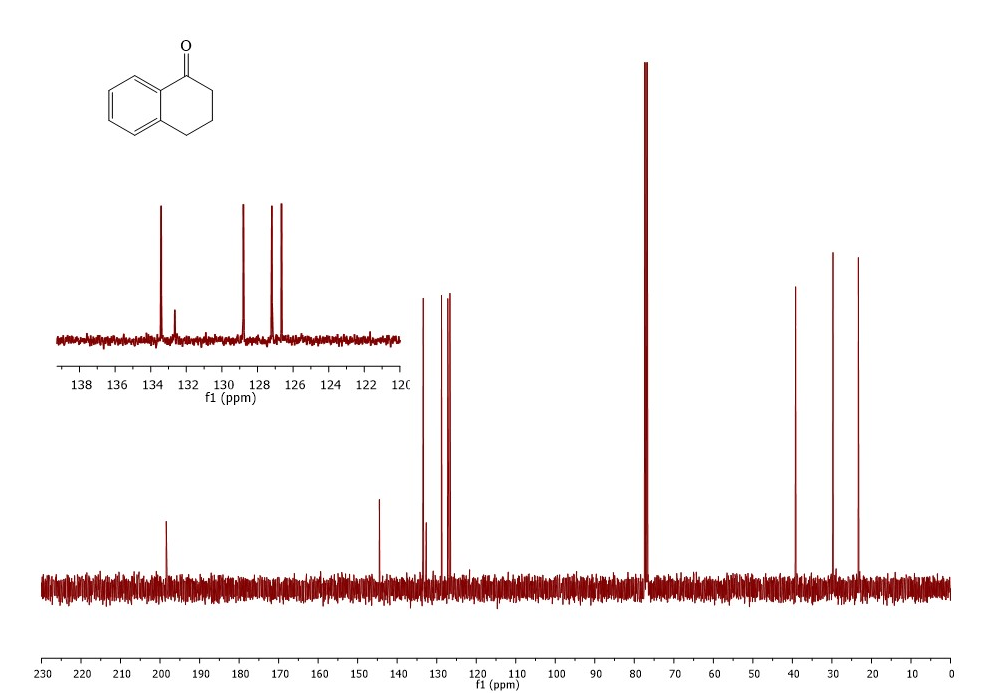
**

**^13^H NMR spectrum of** [**1-tetralone**](https://www.google.com/url?sa=t&rct=j&q=&esrc=s&source=web&cd=5&cad=rja&uact=8&ved=2ahUKEwjr17-9ofTcAhVCzRoKHUGzDnUQFjAEegQIARAB&url=https%3A%2F%2Fpubchem.ncbi.nlm.nih.gov%2Fcompound%2Falpha-Tetralone&usg=AOvVaw3-dUlKOTWyjewjSqsq4bUv)**:**


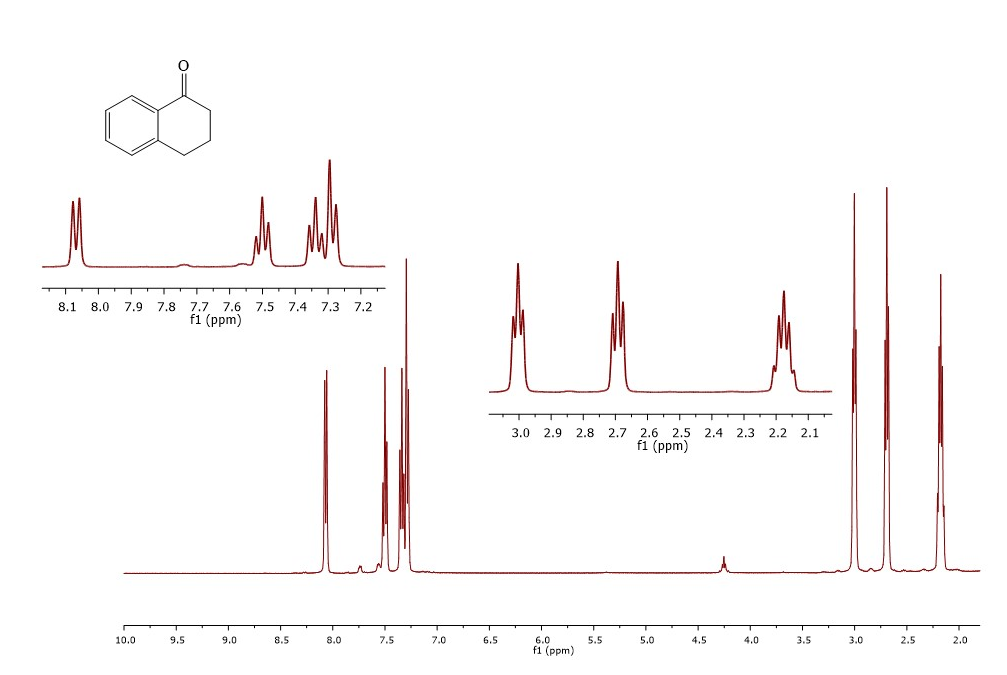


**References**

[1] L. Behrouzi, R. Bagheri, Z. Song, F. Kazemi, B. Kaboudin, M. M. Najafpour, Material Research Express **2019**, 6, 12560.

[2] a) B. Yu, Z. Yang, Y. Zhao, L. Hao, H. Zhang, X. Gao, B. Han, Z. Liu, *Chemistry–A European Journal* **2016**, *22*, 1097-1102; b) N. Jiang, A. J. Ragauskas, *The Journal of organic chemistry* **2007**, *72*, 7030-7033.

[3] G. Zhang, X. Wen, Y. Wang, X. Han, Y. Luan, L. Zheng, C. Ding, X. Cao, *RSC Advances* **2013**, *3*, 22918-22921.

[4] J. Zheng, S. Lin, X. Zhu, B. Jiang, Z. Yang, Z. Pan, *Chemical Communications* **2012**, *48*, 6235-6237.

[5] M. Mogharabi‐Manzari, M. Amini, M. Abdollahi, M. Khoobi, G. Bagherzadeh, M. A. Faramarzi, *ChemCatChem* **2018**, *10*, 1542-1546.

[6] B. Xu, J. P. Lumb, B. A. Arndtsen, *Angewandte Chemie* **2015**, *127*, 4282-4285.

[7] A. K. Mishra, J. N. Moorthy, *Organic Chemistry Frontiers* **2017**, *4*, 343-349.

[8] T. Dohi, K.-i. Fukushima, T. Kamitanaka, K. Morimoto, N. Takenaga, Y. Kita, *Green Chemistry* **2012**, *14*, 1493-1501.

[9] J. Yu, H. Zhao, S. Liang, X. Bao, C. Zhu, *Organic & biomolecular chemistry* **2015**, *13*, 7924-7927.
